# A Behaviorally Informed Mobile App to Improve the Nutritional Quality of Grocery Shopping (SwapSHOP): Feasibility Randomized Controlled Trial

**DOI:** 10.2196/45854

**Published:** 2024-01-11

**Authors:** Carmen Piernas, Charlotte Lee, Alice Hobson, Georgina Harmer, Sarah Payne Riches, Michaela Noreik, Susan A Jebb

**Affiliations:** 1 Nuffield Department of Primary Care Health Sciences University of Oxford Oxford United Kingdom; 2 Department of Biochemistry and Molecular Biology II, Center for Biomedical Research (CIBM), Institute of Nutrition and Food Technology (INYTA), University of Granada Granada Spain; 3 Faculty of Food and Nutrition Sciences Hochschule Niederrhein Mönchengladbach Germany

**Keywords:** swaps, mobile app, supermarket, food purchases, diet, randomized controlled trial, mobile phone

## Abstract

**Background:**

Interventions targeting the nutritional quality of grocery shopping have the potential to help improve diet and health outcomes.

**Objective:**

This study aims to assess the feasibility and acceptability of receiving advice on healthier food purchases through SwapSHOP, a behaviorally informed smartphone app that allows users to scan barcodes of grocery products from the United Kingdom, providing nutritional information and personalized swap suggestions to encourage healthier purchases.

**Methods:**

We randomized adult volunteers in a 6-arm parallel-group controlled feasibility trial. Participants used the SwapSHOP app to record their grocery shopping during a 2-week run-in period and were individually randomized in a 3:1 ratio to either intervention or control arms within 3 strata related to a nutrient of concern of their choice: saturated fat (SFA), sugar, or salt. Participants randomized to the intervention received the SwapSHOP app with a healthier swap function, goal setting, and personalized feedback. Participants in the control group were instructed to use a simpler version of the app to log all their food purchases without receiving any guidance or advice. The primary outcome was the feasibility of progression to a full trial, including app use and follow-up rates at 6 weeks. The secondary outcomes included other feasibility outcomes, process and qualitative measures, and exploratory effectiveness outcomes to assess changes in the nutrient content of the purchased foods.

**Results:**

A total of 112 participants were randomized into 3 groups: SFA (n=38 intervention and n=13 control), sugar (n=40 intervention and n=15 control), and salt (n=5 intervention and n=1 control, not analyzed). The 2 progression criteria were met for SFA and sugar: 81% (30/37) and 87% (34/39) of intervention participants in the SFA and sugar groups, respectively, used the app to obtain healthier swaps, and 89% (68/76) of intervention participants and 96% (23/24) of control participants completed follow-up by scanning all purchases over the follow-up period. The process and qualitative outcomes suggested that the intervention was acceptable and has the potential to influence shopping behaviors. There were reductions of −0.56 g per 100 g (95% CI −1.02 to −0.19) in SFA and −1 g per 100 g (95% CI −1.97 to −0.03) in total sugars across all food purchases in the intervention groups.

**Conclusions:**

People were willing to use the SwapSHOP app to help reduce sugar and SFA (but not salt) in their grocery shopping. Adherence and follow-up rates suggest that a full trial is feasible. Given the suggestive evidence indicating that the intervention resulted in reductions in sugars and SFA, a definitive trial is necessary to target improvements in health outcomes.

**Trial Registration:**

International Standard Randomised Controlled Trial Number ISRCTN13022312; https://doi.org/10.1186/ISRCTN13022312

## Introduction

### Background

Consumption of saturated fat (SFA), sugars, and salt in the United Kingdom and most high-income countries currently exceeds dietary recommendations for good health [1]. These nutrients of concern contribute to the burden of diabetes and cardiovascular disease, either directly or through their effects on blood cholesterol, blood pressure, insulin sensitivity, and body weight [2-8]. Despite decades of nutrition education and efforts to promote healthier behaviors, significant and sustained dietary changes are yet to be observed. Evidence suggests that people need more than general knowledge of dietary recommendations to change behavior [9-11]; however, there is limited evidence for individual-level interventions that are feasible and effective in supporting dietary change at the population level.

Food purchasing is a key antecedent of food consumption, and improving the nutritional quality of food purchases presents a clear opportunity to intervene. Grocery stores account for 71% of the weekly expenditure on food and drinks, including a substantial proportion of foods high in SFA, sugar, and salt [12]. For many foods, there are usually alternatives with less SFA, sugar, or salt, which are functionally similar. This variation can be attributed partly to the natural differences in recipes, such as those found in ready meals or variances in ingredient costs. In addition, it can also be linked to product reformulation driven by consumer demand, such as the introduction of low-fat yogurts, or policy-driven actions, such as changes in soft drink formulations [13].

Systematic reviews have identified some intervention components to support individual dietary change, including providing information, tailored dietary advice, self-monitoring, and personalized feedback [9,14,15]. Specifically, recommending healthier swaps at the point of purchase or as part of individually tailored regular feedback has shown potential to help improve the nutritional quality of grocery shopping [16-20]. Technological advances such as smartphone apps can facilitate this by providing scalable, lower-cost support to help people make healthier choices while shopping.

A systematic review of smartphone apps identified only 2 studies that tested apps that can provide healthier alternatives at the point of choice [21]. Although there is evidence of similar apps in the scientific literature, these previous apps mostly provide healthier swaps to consumers without the option of self-monitoring the quality of their grocery shopping, and many apps have limitations regarding how information is presented to consumers or the limited comprehensiveness of the databases covering the UK food market [22,23]. Our recent proof-of-concept study tested the functionality of the SaltSwap app to help find lower-salt foods when grocery shopping, which included behavioral components such as nutritional information, prompts to lower-salt options, goal setting on swaps, feedback on swaps and salt reduction, and history of swaps [20].

### Objectives

For this study, we have further developed the SaltSwap app into SwapSHOP, a new app that also provides swaps for SFA, sugars, and salt. This study aimed to assess the feasibility and acceptability of receiving dietary advice through the SwapSHOP app. It is a stand-alone intervention that allowed users to scan barcodes of grocery products from major UK stores to obtain nutritional information and suggestions for personalized swaps with lower SFA, sugar, or salt and also enabled users to set goals and monitor their shopping behavior.

## Methods

### Study Design

This was a prospectively registered (International Standard Randomised Controlled Trial Number: ISRCTN13022312) randomized 6-arm parallel-group controlled feasibility trial conducted in the United Kingdom. After giving written informed consent to participate in the study and completing screening and baseline assessment, participants entered a 2-week run-in period where they used a basic version of the SwapSHOP app (no swap or behavioral functionality shown) to record their grocery shopping. They were individually randomized to 1 of the intervention arms or control following a 3:1 ratio (intervention: control) within 3 strata related to a nutrient of concern of their choice (SFA, sugar, or salt). Individuals participated in the study for 6 weeks from screening to the final follow-up.

### Ethical Considerations

This study was reviewed and approved by the University of Oxford Medical Sciences Interdivisional Research Ethics Committee (R67216/RE001). Informed consent was obtained from all the participants.

### Participants and Procedures

Participants were recruited between April and October 2021 through the community (eg, word of mouth) and through social networks and media, online newsletters and newspapers, and electronic mailing lists. Participants were eligible if they were aged >18 years, were willing and able to give informed consent, were English speaking and able to understand the demands of the study, were looking for support to improve their diet, owned a smartphone with access to the internet and an email account, were willing to download and use a smartphone app to scan and track their grocery shopping for the 6 weeks during their participation in the study, were responsible for at least some of their household grocery shopping, and shopped regularly (eg, at least once a week in a physical store or online at any of the 6 major food retailers in the United Kingdom: Tesco, Sainsbury’s, Waitrose, Asda, Morrisons, or Iceland). People were not eligible if they were already on a clinician-supervised diet or a restricted diet, were currently using apps to monitor the quality of their food shopping (excluding apps to track and monitor food intake), were currently participating in another study aimed at dietary change or asking them to change the way they shop for food, or were planning on going away from home (holiday or other) for >2 consecutive weeks following enrollment in the study.

Eligible participants were invited to complete a baseline web-based questionnaire that collected demographic information, relevant self-reported medical history, and details about their shopping behaviors. Participants also reported the nutrient (SFA, sugar, or salt) they were most concerned about in their diet, which was used as a stratification factor during the randomization process. Participants then entered a 2-week run-in period during which they were asked to record all their food purchases, either by scanning the barcode of purchased grocery products or manually inputting what was purchased using a simpler version of the SwapSHOP app. Only participants who completed this task with good engagement with the app were randomized. Good engagement was defined as scanning products in at least 2 shops, each with products from at least 3 different predefined food categories (eg, fresh meat, chilled ready meals, and soft drinks), and scanning products from ≥5 of the food categories across the 2 weeks.

### Randomization and Blinding

After the 2-week run-in period, participants were individually randomized to either an intervention arm or control following a 3:1 ratio (intervention:control) within each stratum depending on their chosen nutrient of concern (sugar, salt, or SFA). Participants who did not complete the baseline data collection were not randomized. Randomization was performed with a computer using a random allocation sequence (concealed to the investigators) and was stratified by the nutrient of concern using block randomization with block sizes of 4 and 8.

It was not possible to blind the participants to the intervention because of the nature of the intervention; however, randomization was performed remotely via computer-generated randomization, and the researchers were not aware of the treatment group until after randomization was complete.

### Intervention

Participants randomized to the intervention arms were able to access an enhanced version of the SwapSHOP app, which included a healthier swap function, goal setting, and personalized feedback. Participants scanned the barcode of grocery products to receive nutritional information about the product using the UK traffic light system and were presented with healthier alternatives or swaps that were lower in SFA, sugar, or salt (depending on group allocation). The swaps that appeared after scanning the original product were chosen from a list of products within the same food category, which were ranked from a larger to a smaller reduction in the specific nutrient that was initially chosen. Participants could also set goals for the number of swaps they wanted to make in each shopping trip and record the products they swapped in the app. The app had specific functions to provide feedback on the achievement of their goals and feedback on the overall nutrient reduction achieved by making swaps. The app also had a section to display the ranking of all purchased foods contributing the most to each nutrient of concern (Figure S1 in Multimedia Appendix 1).

The SwapSHOP app included a comprehensive database of >70,000 grocery products available in January 2021 within major UK grocery stores (including Morrisons, Sainsbury’s, Tesco, Waitrose, Iceland, and Asda) [24] and a bespoke system for categorizing products and selecting suitable alternative swap suggestions. SwapSHOP was based on a previous version developed exclusively for salt, and its theoretical basis is grounded in the Behavior Change Wheel [20,25], a framework that integrates the elements of capability, opportunity, and motivation, which are key for behavior change. A range of behavior change techniques from the taxonomy groups *goals and planning* and *feedback and monitoring* that were incorporated into this intervention have been associated with successful dietary change [9,26].

To enable assessment of changes in the nutritional composition of the shopping baskets, participants received weekly reminders to continue scanning and recording all their purchased products for the remaining 4 weeks of follow-up (with a minimum of 2 full weeks of grocery shopping data during the follow-up period).

### Comparator

Participants randomized to the control arm were asked to continue using the simpler version of the app with no product nutrition information, swaps, or behavioral components, solely to record all their food purchases as part of the outcome assessment at the end of the 6-week follow-up.

### Outcome Measures

#### Primary Outcomes

The primary objective of this study was to determine the feasibility of a larger study to evaluate the effectiveness of an app that offers healthier swaps while grocery shopping to improve the nutritional quality of food purchasing with respect to sugar, salt, or SFA. The primary outcomes were prespecified progression criteria as follows:

1. App use: at least 70% of participants in the active intervention group use the app to obtain swaps on at least 1 occasion by the end of the second week after randomization.
2. Follow-up rate: at least 60% of participants in total (intervention and control) complete follow-up by scanning all their purchases for a minimum of 2 weeks over the entire follow-up period (4 weeks).

#### Secondary Outcomes

Feasibility outcomes included (1) recruitment rates: total recruited (including number signed up, eligible, consented, and randomized), (2) time needed for recruitment of the final sample, (3) outcome reporting: number of participants who failed to scan their purchases for a minimum of 2 weeks during follow-up, and number of participants who failed to complete the end-of-study questionnaires.

#### Process Evaluation and Qualitative Outcome Measures

A summary of app-related use (within-app automatic recording) and acceptability measures was collected through the end-of-study questionnaires at follow-up: (1) average number of shopping trips where the app was used to scan products to obtain a swap per week; (2) number of occasions the app was used to scan products for a swap per trip; (3) number of swaps made overall per week and per shopping trip; (4) nutrient (SFA, sugar, and salt) reduction per swap made; (5) use of specific functionality, for example, goal setting and feedback; (6) end-of-study questionnaires with free text to understand app use and acceptability of the swaps, app functionality (eg, if app scans most products), and if this prompted other behaviors such as reading nutrition labels.

#### Exploratory Effectiveness Outcomes

Measures included changes in the nutrient content (SFA and sugar in grams per 100 g) of household food purchases recorded in the app between baseline and follow-up in the intervention group compared with the control group.

### Statistical Analysis and Sample Size

This study was planned as a feasibility study. A sample size of 120 (n=approximately 30 participants per intervention group and n=approximately 10 participants per control group) was prespecified as sufficient to enable testing of the trial methodology and outcome measures using parametric statistical models. The protocol was prospectively registered (International Standard Randomised Controlled Trial Number: ISRCTN13022312).

Baseline characteristics overall and by trial arm were summarized as means and SDs for continuous variables and counts and percentages for categorical variables. Baseline characteristics were coded as age (years), gender (man, woman, other, or not specified), ethnic group (Asian, Black, White, mixed, or other), education (no formal qualifications, secondary education, or higher education), income (>£15,000, £15,000-£24,999, £25,000-£39,999, £40,000-£75,000, and >£75,000; GBP £1=US $1.38), household size (1, 2-4, and ≥5), grocery shopping habits (eg, spending >£25) on groceries more than once a week, once a week, once a fortnight, once a month, and less than once a month), and relevant self-reported health history (eg, concerns related to weight, high blood pressure, diabetes, and heart disease).

Descriptive statistics were used to present the primary and secondary outcomes using all available data, regardless of whether the participants completed the trial or withdrew.

For the exploratory effectiveness measures, we used data from products purchased over the 2 weekly periods recorded at the beginning and end of the trial, with available information on weight or volume as well as the nutrient content (grams per 100 g) to estimate changes in SFA and sugar for food purchases recorded in the app. The excluded items in this analysis included those categorized as fresh fruits, vegetables, or with no or minimal nutrient content (eg, sugar-free gum) as well as products that were manually entered in the app or those missing nutrient or volume information. We used linear regression models to investigate changes in the nutrient content of food purchases between baseline and follow-up in the intervention and control groups. Tests for linear regression assumptions were conducted and met. The main models included adjustment for baseline values of the dependent variable. Potential confounding because of imbalance in baseline characteristics was investigated, and the following variables were adjusted in sensitivity analyses: age (years), gender (man, woman, other, or not specified), ethnic group (Asian, Black, White, mixed, and other), and income (>£15,000, £15,000-£24,999, £25,000-£39,999, £40,000-£75,000, and >£75,000). StataSE (version 16; StataCorp) was used for all the analyses. A *P* value of <.05 was set to denote statistical significance.

A descriptive analysis of the qualitative outcome measures was conducted using the method by Braun and Clarke [27] for thematic analysis in NVivo 1 software (Lumivero). Each response was line-by-line coded, and codes were inductively constructed based on the aim of the study. We then organized codes into subthemes using the One Sheet of Paper technique and produced top-level themes [28].

### Patient and Public Involvement

The SwapSHOP app was based on a previous version developed exclusively for salt reduction [20], which was conceived and tested using input from a patient and public involvement panel. People told us that they would also value an app to help change other aspects of their diet. The current app was also beta tested by members of the public recruited from the community.

## Results

### Participant Characteristics

**Figure 1:**
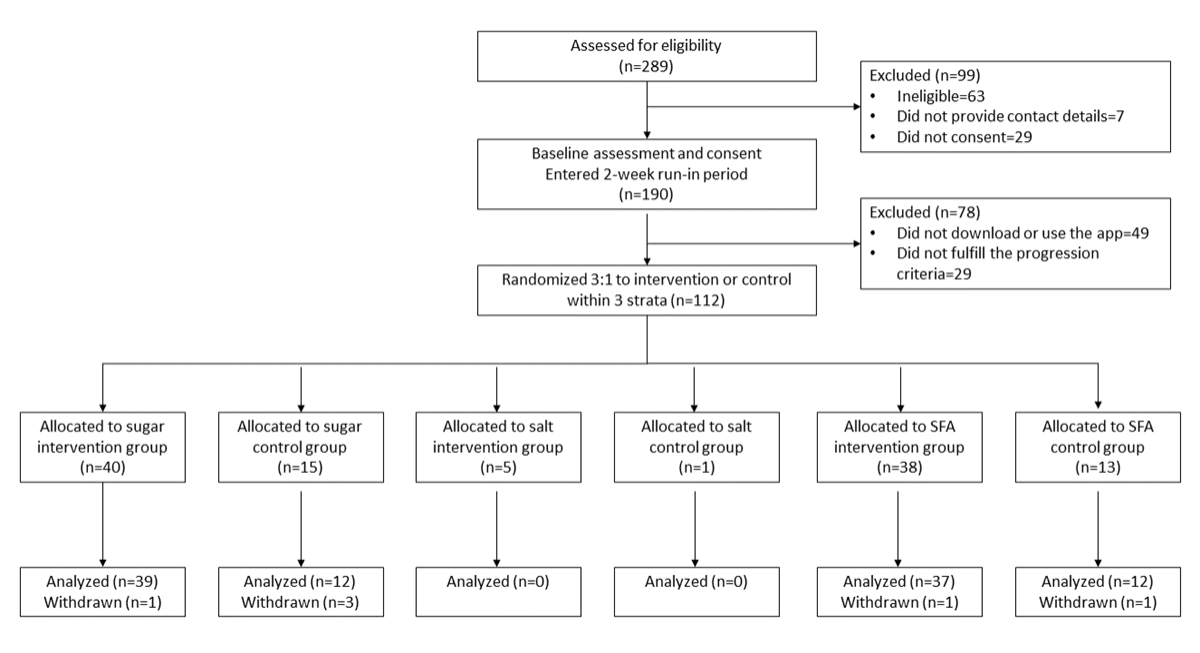
CONSORT (Consolidated Standards of Reporting Trials) diagram. SFA: saturated fat.

Study participants were recruited between April and October 2021. Of the 289 interested participants, 190 (65.7%) were eligible, provided consent, and entered the 2-week run-in period. Of these 190 participants, 78 (41%) were excluded because they did not download or use the app (n=49, 63%) or did not fulfill the progression criteria based on their engagement with the app (n=29, 37%). The final sample of 38.8% (112/289) of participants successfully completed the task and were randomized (Figure 1). A total of 51 participants were randomized to the SFA group (n=38 to intervention and n=13 to control), 55 participants were randomized to the sugar group (n=40 to intervention and n=15 to control), and 6 participants were randomized to the salt group (n=5 to intervention and n=1 to control). Of the randomized participants, 100 (89%) completed the study and were analyzed as follows: 49 (96%) in the SFA group and 51 (93%) in the sugar group. A total of 2 participants in the SFA group and 4 participants in the sugar group were withdrawn from the study and excluded from the analysis because their data indicated fraudulent activity (eg, fake phone numbers and implausible shopping patterns). Data from 6 participants who were randomized to the salt group were not analyzed, as this group did not reach the target sample by the end of the recruitment period.

Participants in the analytic sample were, on average, aged 35 (SD 12) years, mostly women (80/100, 80%) of White ethnicity (79/100, 79%), and from higher education backgrounds (73/100, 73%; Table 1). Participants mostly lived in households with 2 to 4 other members, shopped once (61/100, 61%) or more than once a week (26/100, 26%), shopped mostly in larger grocery stores (89/100, 98%), or shopped on the internet (39/100, 39%). Most participants looked at SFA or sugar information on nutrition labels always, often, or sometimes and approximately half of the sample (54/100, 54%) reported concerns related to weight among the relevant medical conditions.

**Table 1:** Baseline characteristics of participants in the analytic sample^a^.

			Total population (n=100)		Sugar group				Saturated fat group		
					Control (n=12)		Intervention (n=39)		Control (n=12)		Intervention (n=37)
Age (y), mean (SD)			35 (12)		36 (8)		35 (10)		32 (10)		37 (16)
Gender (woman), n (%)			80 (80)		9 (75)		33 (85)		9 (75)		29 (78)
**Ethnic group, n (%)**											
	Black or Asian	9 (9)		1 (8)		6 (15)		0 (0)		2 (5)	
	White	79 (79)		10 (83)		27 (69)		12 (100)		30 (81)	
	Mixed, other, or not specified	12 (12)		1 (8)		6 (15)		0 (0)		5 (14)	
**Education, n (%)**											
	No qualifications or not specified	2 (2)		0 (0)		0 (0)		0 (0)		2 (5)	
	Secondary education	25 (25)		0 (0)		12 (31)		3 (25)		10 (27)	
	Higher education	73 (73)		12 (100)		27 (69)		9 (75)		25 (68)	
**Individual income^b^, n (%)**											
	<£15,000	28 (28)		3 (25)		9 (23)		2 (17)		14 (38)	
	£15,000-£24,999	24 (24)		4 (33)		10 (26)		2 (17)		8 (22)	
	£25,000-£39,999	31 (31)		4 (33)		10 (26)		8 (67)		9 (24)	
	£40,000-£75,000	11 (11)		1 (8)		6 (15)		0 (0)		4 (11)	
	>£75,000	1 (1)		0 (0)		1 (3)		0 (0)		0 (0)	
**Household size, n (%)**											
	1	29 (29)		2 (17)		11 (28)		3 (25)		13 (35)	
	2-4	64 (64)		8 (67)		24 (62)		9 (75)		23 (62)	
	≥5	7 (7)		2 (17)		4 (10)		0 (0)		1 (3)	
**Frequency of grocery shopping (≥ £25 per trip), n (%)**											
	More than once a week	26 (26)		7 (58)		8 (21)		5 (42)		6 (16)	
	Once a week	61 (61)		4 (33)		26 (67)		7 (58)		24 (65)	
	Once a fortnight	11 (11)		1 (8)		3 (8)		0 (0)		7 (19)	
	Once a month	1 (1)		0 (0)		1 (3)		0 (0)		0 (0)	
	Less than once a month	1 (1)		0 (0)		1 (3)		0 (0)		0 (0)	
**Type of grocery shop usually visited, n (%)**											
	Supermarkets	98 (98)		12 (100)		38 (97)		11 (92)		37 (100)	
	Online supermarkets	30 (39)		5 (42)		11 (28)		4 (33)		10 (27)	
	Corner shop or convenience store	32 (32)		1 (8)		14 (36)		3 (25)		14 (38)	
	Greengrocers or fruit and vegetable shop	12 (12)		3 (25)		5 (13)		0 (0)		4 (11)	
	Butchers or meat market	11 (11)		0 (0)		6 (15)		2 (17)		3 (8)	
	Other fresh food markets	17 (17)		4 (33)		7 (18)		0 (0)		6 (16)	
**Looking at salt in nutrition labels, n (%)**											
	Always or often	14 (14)		2 (17)		3 (8)		5 (42)		4 (11)	
	Sometimes	24 (24)		3 (25)		12 (31)		1 (8)		8 (22)	
	Rarely or never	62 (62)		7 (58)		24 (62)		6 (50)		25 (68)	
**Looking at sugar in nutrition labels,** **n (%)**											
	Always or often	42 (42)		2 (17)		19 (49)		7 (58)		14 (38)	
	Sometimes	30 (30)		6 (50)		10 (26)		2 (17)		12 (32)	
	Rarely or never	28 (28)		4 (33)		10 (26)		3 (25)		11 (30)	
**Looking at fat in nutrition labels, n (%)**											
	Always or often	29 (29)		1 (8)		6 (15)		8 (67)		14 (38)	
	Sometimes	44 (44)		7 (58)		21 (54)		1 (8)		15 (41)	
	Rarely or never	27 (27)		4 (33)		12 (31)		3 (25)		8 (22)	
**Relevant health conditions, n (%)**											
	Concerns related to weight	54 (54)		7 (58)		18 (46)		5 (42)		24 (65)	
	High blood pressure	5 (5)		0 (0)		2 (5)		0 (0)		3 (8)	
	Diabetes	11 (11)		2 (17)		4 (10)		1 (8)		4 (11)	
	Heart disease	4 (4)		0 (0)		1 (3)		1 (8)		2 (5)	
**Relevant current medications, n (%)**											
	High blood pressure	4 (4)		0 (0)		1 (3)		0 (0)		3 (8)	
	Diabetes	6 (6)		0 (0)		2 (5)		1 (8)		3 (8)	
	Heart disease	1 (1)		0 (0)		0 (0)		1 (8)		0 (0)	

^a^The number of participants recruited for the salt group did not reach the target sample size. Data from the participants in this group were not analyzed further.

^b^GBP £1=US $1.38.

### Primary Outcomes

The number of participants recruited for the salt group did not reach the target sample size despite additional recruitment attempts, suggesting that a full-scale trial for salt reduction would not be feasible. Data from the participants in this group were not analyzed further.

For participants randomized to the SFA and sugar groups, the 2 progression criteria were met above the set thresholds (Table 2). Overall, most intervention participants (30/37, 81% in the SFA group and 34/39, 87% in the sugar group) used the app to obtain swaps on at least 1 occasion by the end of the second week after randomization. In addition, 89% (68/76) of the intervention participants and 96% (23/24) of the control participants completed follow-up by scanning all purchases for a minimum of 2 weeks over the entire follow-up period.

**Table 2:** Primary and secondary outcomes—progression criteria and feasibility outcomes^a,b^.

			Total population, n (%)							Sugar group, n (%)					Saturated fat group, n (%)		
			Total (N=100)		Control (n=24)		Intervention (n=76)		Control (n=12)			Intervention (n=39)		Control (n=12)			Intervention (n=37)
**Primary outcomes—progression criteria**																	
	Participants used the app to obtain healthier swaps on at least 1 occasion by the end of the second week after randomization	N/A^c^		N/A		64 (84)		N/A			34 (87)		N/A			30 (81)	
	Participants completed follow-up by scanning all purchases for a minimum of 2 weeks over the entire follow-up period	91 (91)		23 (96)		68 (89)		11 (92)			36 (92)		12 (100)			32 (86)	
**Secondary outcomes—feasibility outcomes**																	
	Signed-up participants	289 (100)		N/A		N/A		N/A			N/A		N/A			N/A	
	Eligible participants	226 (78)		N/A		N/A		N/A			N/A		N/A			N/A	
	Consented participants	197 (68)		N/A		N/A		N/A			N/A		N/A			N/A	
	Completed baseline assessments	141 (49)		N/A		N/A		N/A			N/A		N/A			N/A	
	Randomized participants	106 (100)		28 (100)		78 (100)		15 (100)			40 (100)		13 (100)			38 (100)	
	Failed to complete follow-up or end-of-study questionnaire	16 (15)		0 (0)		16 (21)		0 (0)			9 (23)		0 (0)			7 (18)	

^a^Percentage of eligible, consented, and completed participants was calculated based on the number of signed-up participants originally; the number of randomized participants (total population) excluded those randomized to the salt group, as these were not analyzed.

^b^The number of participants recruited for the salt group did not reach the target sample size. Data from the participants in this group were not analyzed further.

^c^N/A: not applicable.

### Secondary Outcomes

The secondary outcomes provided evidence that a larger trial aiming at reducing sugars or SFA would recruit enough participants that adhere to the trial procedures (ie, completing baseline assessments) within the set time frames and that 84% (16/100 failed to complete follow-up) of those randomized would complete follow-up assessments.

### Process Evaluation and Qualitative Outcomes

Participants randomized to the SwapSHOP intervention used the app regularly (Table 3). The average number of shopping trips where the app was used to obtain a swap was 5 in the SFA group (92% of shopping trips) and 5.4 in the sugar group (83% of shopping trips). The average number of occasions when the app was used to scan products for a swap in each shopping trip was 2.5 times in the SFA group and 3.3 in the sugar group. Overall, participants set goals averaging approximately 2 swaps per shopping trip. The results showed that individual product swaps were associated with an average reduction in total sugars (−12.5 g of sugar per 100 g, SD 6.97) or in SFA (−4 g of SFA per 100 g, SD 2.16) in the sugar and SFA groups, respectively.

**Table 3:** Secondary outcomes—process evaluation measures among participants using the swap function^a,b^.

	Sugar group (n=34), mean (SD)	Saturated fat group (n=30), mean (SD)
Average number of shopping trips where the app was used to obtain a swap	5.41 (5.14)	5.07 (3.39)
Percentage of total shopping trips where the app was used to obtain a swap	83.15 (24.74)	91.62 (18.83)
Occasions the app was used to scan products for a swap per shopping trip	3.31 (2.30)	2.49 (1.94)
Average swap goals set in the app per shopping trip	2.14 (1.15)	2.01 (1.54)
Sugar reduction per swap (grams per 100 g)	−12.47 (6.97)	−0.48 (6.70)
Saturated fat reduction per swap (grams per 100 g)	−1.62 (2.57)	−4.02 (2.16)

^a^Sugar and SFA reduction per swap per 100 g were calculated as the average change in nutrient per 100 g of product across all swaps made during the intervention period.

^b^The number of participants recruited for the salt group did not reach the target sample size. Data from the participants in this group were not analyzed further.

The results of the qualitative research relating to the acceptability of the app, the feedback and swaps provided through the app, and the usefulness and comprehensiveness of the app were summarized into 4 themes (Table S1 in Multimedia Appendix 1). Overall, most participants shared positive experiences of the intervention, noting that the app was helpful and the swap suggestions were acceptable. They valued the novelty of the app and the traffic light food labeling system, which encouraged them to read food labels. The key barriers to usability were that some grocery stores were not supported by the app and that there was poor product coverage within the app database. Although participants could manually enter their food purchases to find alternatives, they reported that this was time consuming and a barrier to engagement. Some participants also found it difficult to locate the suggested swaps in the store or were frustrated that the suggested swaps were not available in the store or that the product database in the app did not include own-label products in some stores. One significant obstacle to accepting the intervention was the specificity of certain swap suggestions. These suggestions were not always direct replacements or tailored to personal or household dietary preferences, which made it challenging for shoppers to act upon the prompts. In other instances, some participants found it challenging to reduce one nutritional component if it involved an increase in another, and they expressed a desire for a swap suggestion that recognized the overall healthiness of the product. Participants suggested that more information on the fiber composition of swaps, recommended portion sizes, and price comparison would inform their purchasing behavior. Most participants noted that other visual self-monitoring techniques would improve engagement with the intervention.

### Exploratory Effectiveness Outcomes

Baseline and follow-up data on food purchases with available information on volume and nutrient content were available for 86% (44/51) of the participants in the sugar group and 82% (40/49) of the participants in the SFA group. This analysis included all food purchases recorded in the app but excluded purchases that were entered manually (22%) or those with no, minimal, or missing nutritional information; 16%). The degree of missingness in the food purchasing data was comparable between the groups (manually entered products: 23% intervention vs 19% control group and missing nutrient information: 15% intervention vs 16% control group).

There was evidence of changes in the intended direction in both the intervention groups (Table 4). The sugar group reduced total sugars in their grocery purchases by −1 (95% CI −1.97 to −0.03) g/100 g, whereas the control group reduced total sugars in their grocery purchases by 0.32 (−1.47 to 2.11) g/100 g, though the differences between groups were not significant (−0.68, 95% CI −1.94 to 0.58 g/100 g; *P*=.28 adjusted for baseline values). The SFA group reduced total SFA in food purchases by −0.56 (95% CI −1.02 to −0.19) g/100 g, and the control group increased total SFA in food purchases by 0.52 (95% CI −0.19 to 1.22) g/100 g, with a significant between-group difference of −1.05 (95% CI −1.83 to −0.27) g/100 g and *P*=.009 adjusted for baseline values. These results were robust in sensitivity analyses adjusted for age, gender, ethnicity, and income (Table S2 in Multimedia Appendix 1).

**Table 4:** Secondary outcomes—exploratory effectiveness measures^a,b^.

			Baseline, mean (SD)		Follow-up, mean (SD)		Change, mean (95% CI)		Change adjusted^b^, mean (95% CI)		Between-group difference^b^, intervention vs control		
											Mean (95% CI)		*P* value
**Purchased sugar (g/100 g)**													
	Sugar group (n=34)	5.13 (2.63)		4.13 (2.34)		−1.00 (−1.97 to −0.03)		−0.86 (−1.67 to −0.05)		−0.68 (−1.94 to 0.58)		.28	
	Control (n=10)	4.26 (1.49)		4.58 (1.86)		0.32 (−1.47 to 2.11)		−0.18 (−1.14 to 0.79)		N/A^c^		N/A	
**Purchased SFA^d^ (g/100 g)**													
	SFA group (n=28)	2.13 (1.18)		1.58 (0.98)		−0.56 (−1.02 to −0.10)		−0.55 (−0.89 to −0.22)		−1.05 (−1.83 to −0.27)		.009	
	Control (n=12)	2.10 (0.84)		2.61 (1.22)		0.52 (−0.19 to 1.22)		0.50 (−0.20 to 1.20)		N/A		N/A	

^a^Linear regression adjusted for baseline values.

^b^The number of participants recruited for the salt group did not reach the target sample size. Data from the participants in this group were not analyzed further.

^c^Not applicable.

^d^SFA: saturated fat.

## Discussion

### Principal Findings

Participants in this study were willing and able to use the SwapSHOP app as intended, and the research methods ran as planned, with high levels of adherence and follow-up recorded. This provided preliminary evidence of effectiveness to support dietary change to lower the intake of sugars and SFAs. There was little evidence that this general population sample was motivated to reduce salt intake using the SwapSHOP app. Further improvements to the app, especially enhancing the coverage of product data and the specificity of the swap algorithm, are needed to provide a higher quality user experience.

### Comparison With Prior Work

Previous studies have shown the potential of instore swaps to support healthier choices, but there is very little evidence from interventions involving habitual shopping in physical stores. A quasi-experimental study evaluated the impact of the Change4Life Smart Swaps campaign to promote changes to lower fat or sugar foods when grocery shopping, showing that a higher percentage of participants in the intervention group reported choosing healthier options at the end of the study [29]. A total of 3 randomized controlled trials used a smartphone app to help reduce salt intake by promoting lower-salt swaps at the point of choice, showing changes in purchased salt in the intended direction but without evidence of changes in salt intakes [20,23,30]. Our previous study conducted in primary care settings provided individually tailored regular feedback on food shopping and offered lower-SFA swaps to patients with raised low-density lipoprotein cholesterol. The study showed modest but nonsignificant reductions in SFA consumption and SFA in purchased foods [18]. Other randomized controlled trials conducted in online retail environments, both real and experimental platforms, have shown that offering healthier swaps at the point of choice helps improve the nutritional quality of food shopping [16,17,19].

This SwapShop intervention showed promising signs of early effectiveness, with observed reductions of −1 g/100 g (95% CI −1.97 to −0.03) in total sugars and −0.56 (95% CI −1.02 to −0.19) in SFA in all food purchases in their respective intervention group, though this feasibility study was not powered to detect an effect in purchased nutrients. Data also show that smartphone app use is increasing across a wide range of demographic and age groups [31], and consumers are showing an increasing interest in healthier options [32]. However, although the app may be a useful tool for promoting healthier food choices through personalized advice and support, it is unlikely to be sufficient to achieve dietary recommendations. It is plausible that the impact could be enhanced by interventions to encourage product reformulation to offer greater availability of healthier alternatives. Moreover, price, promotions, positioning, and availability strategies within supermarkets have all been found to be major determinants of food choices [33-36], and these structural interventions are likely to be complementary.

### Strengths and Limitations

This study’s strength lies in its randomized design and the process evaluation used to investigate the intervention’s pathway to impact. The qualitative components helped to provide context-specific information about the usability of the app and the acceptability of the swap suggestions. The SwapSHOP app had undergone extensive testing before this trial, given that it is an enhanced version of a previously tested app (SaltSwap) that was specifically designed to help people with hypertension choose lower-salt foods when grocery shopping in combination with face-to-face advice from a health care professional [20,37]. Furthermore, beta testing helped refine the app in line with the intended user feedback. SwapSHOP incorporated several behavioral elements with proven evidence to support dietary change [9,14,15], allowing people to set goals to swap to foods, lower the nutrients of concern, identify major sources of SFA and sugar in their shopping, and provide feedback on achievement of their goal as well as on the overall nutrient reduction achieved through making swaps. A systematic review of interventions using apps to support dietary behavioral improvements suggested greater benefits of multicomponent interventions compared with single-component interventions [38]. Unlike SwapSHOP, the 2 other existing smartphone apps provided information on healthier swaps but did not offer the option of self-monitoring the nutritional quality of food shopping and had limitations regarding how information is presented to consumers or the limited comprehensiveness of the databases covering the UK food market [21,22].

Although the SwapSHOP app included the major UK grocery retailers, participants particularly reported issues related to the product database, the limited range of UK grocery stores, and the improvements needed in the swapping function to create a list of healthier options that are similar to the original product and to accommodate dietary preferences. These aspects must be addressed in future versions of the app to maximize usability and acceptability. Purchased products that were recorded manually by the participants or had missing nutrient information were excluded from the analysis of changes in total purchases, limiting the robustness of the exploratory effectiveness outcome results, although this should not differ by trial arm.

Another limitation is that the study recruited a small, self-selected sample of people who were motivated to take action to improve their diet quality. A large proportion of participants reported receiving higher education and living in less deprived geographical areas than the national average [39]. A lower socioeconomic status is related to poorer dietary quality and health outcomes; hence, the observed results may not represent a wider population with lower adherence to dietary recommendations [40]. Ethnicity is also related to food choice [41]. Although this study mostly included people of White backgrounds, the app database included a wide range of products available in the UK market, thus covering different dietary preferences that apply to other ethnic groups.

### Conclusions

SwapSHOP is a behaviorally informed smartphone app that allows users to scan barcodes of grocery products from major UK supermarkets, providing tailored nutritional information and suggesting personalized swaps to support dietary change. This study provided evidence of feasibility as a stand-alone intervention to support motivated individuals wanting to reduce their SFA or sugar intake as well as preliminary evidence of effectiveness to support healthier food purchases. Given the low cost and scalability of this intervention, after further refinement of the app technology and expanded market coverage, a definitive trial is warranted to assess the potential of this tool to improve health outcomes.

## Appendix

Multimedia Appendix 1 Supplementary tables and figures.

Multimedia Appendix 2 CONSORT eHEALTH checklist (V 1.6.1).

## Abbreviations

SFA: saturated fat

## Data Availability

The data sets generated during and/or analyzed during this study are available on reasonable request. Access to the study data and the statistical code will be reviewed and granted upon request by the Nuffield Department of Primary Care PRimDISC committee (primdisc@phc.ox.ac.uk)
