# Supplementary material for: A Behaviorally Informed Mobile App to Improve the Nutritional Quality of Grocery Shopping (SwapSHOP): Feasibility Randomized Controlled Trial
Source: JMIR Mhealth Uhealth. 2024 Jan 11;12:e45854. doi: 10.2196/45854 (PMC10811579; doi:10.2196/45854)
Supplement: Multimedia Appendix 1 [file mhealth_v12i1e45854_app1.docx]

Multimedia appendix 1

Table S1 – Themes and quotes of participants’ reported usability and acceptability of the SwapSHOP intervention

| Theme | Sub-theme | Description | Quotes |
| --- | --- | --- | --- |
| Helpful | User-friendly app | Participants described the app as easy to follow. They valued (1) scanning product barcodes, which they reported as fast and easy; (2) the traffic light labelling system, especially on products not marked by colours; and (3) the accompanying pictures, which made it easy to identify the swap suggestion. | “Easy colour coded guidelines so I could quickly scan through the sugar levels of multiple products.”  “I really like the fact when you're searching up for the items, it shows up along with the picture and a brief description so that was super helpful in making sure that I was putting the right items into the purchase list.” |
|  | Novel educational mechanism | Participants reported that the app was enjoyable and a novel way to access nutritional information. For some, the information provided was surprising and prompted them to start paying attention to food labels. | “I liked the swap suggestions – it has really helped me to be more aware of sugar intake with my shopping.”  “It did make you think about the salt sugar and fat content before purchase.” |
|  | Introduced novel swap suggestions | The swap suggestions were often perceived as useful because it introduced participants to new and healthier product ideas that they had not previously considered. | “The different swaps offered I find very useful as they showed products that you would never of thought of purchasing or knew they existed in the shop you were shopping in.”  “It helped me to have a better idea about the sugar content of the food i was buying, and introducing me to alternative products i did not think of purchasing before. It also helped to understand which products have more sugar.” |
| Unhelpful | Supermarkets and products that were not supported by the app | Participants reported poor supermarket or product coverage. Some items were unavailable for scanning because (1) the supermarket was unsupported by the app; or (2) the product was not listed, particularly own-brand items.  They described using the manual entry function. They expressed concern that this was effortful and time-consuming, which negatively impacted their enthusiasm to engage with the intervention. | “Not all products I was buying could be successfully scanned with the app or some products were not on the product list either, which made it a bit painful to log in my entire weekly shopping.”  “I didn't have a great success rate with scanning items in store – often [I] had to input manually which is rather time consuming and means you are stood faffing with your phone while other shoppers are trying to get to the displays.” |
|  | Specificity of the swap suggestions | Participants expressed concerns that the swaps were not like-for-like, whereas others noted that the swap suggestions did not account for personal / household dietary requirements. For some, this meant they could not act on the suggestion. | “I think the swapping was a bit complicated and unhelpful: for example, if I wanted to buy a ready-made savoury pastry, it would suggest I swap it for a jar of savoury spread, like pesto.”  “Not taking into account dietary requirements or preferences: Some of the people in my household are vegan, and most of the time the lower fat swaps offered were not vegan, thus we couldn't make them.” |
| Changes | Improve supermarket and product coverage | Participants said that they shopped in more than one supermarket. They wanted more stores and products to be included in the app, which would open up possibilities to act on a swap suggestion and boost engagement. | “Maybe to introduce other supermarkets on the app, as for example, Lidl, Aldi, etc. I do my shopping in different supermarkets and I would find useful to find low sugar alternatives in a[n]y place.”  “I couldn't find alot of my shopping so had to manually enter alot which became time consuming and people might give up.” |
|  | App interface and function | There were some difficulties in navigating the swap function, which interrupted their shopping experience. Other participants expressed concerns with the interface of the app (i.e., layout and brightness). | “I realised when I made the swap, I have to go back to the previous screen to continue to choose other product had purchased, sometimes that is a bit inconvenient.  “... make the shopping for swaps simpler – rather than having to find and accept a number of swaps before shopping for other items, allow people to just shop and see alternatives as appropriate (i do not have time to go back and forth in store trying to locate specific items).” |
| Improvements | Overall healthfulness of their grocery shop | Participants described compromising on one nutritional component against another. To achieve a more balanced diet, they recommended an overall health rating of each food item and their shopping basket.  Others suggested information on fibre intake, portion sizes and price difference would be helpful to inform their purchasing behaviour. | “Overall health rating of particular foods? [S]ome items may be low in sugar but high in fat/salt, etc so overall may be more unhealthy than a product with a more balanced sugar/fat/salt content.”  “Salt, sugar and fat are the main dietary factors in health but I personally would find it helpful to see swaps for fibre content in food.”  “Showing me the cost of the swaps! I'm not going to buy something that's slightly lower sugar if it's double the price.” |
|  | Goal setting and self-monitoring features | Participants suggested that engagement with the intervention could be supported with behavioural techniques (i.e., goal setting and self-monitoring). They recommended that this would be key to sustain adherence. | “A meal planner would be useful – set up what meals you want to have each day and where you shop, the app would then list the healthy products to speed up the shopping trip and confidence it was healthier before you go.”  “Have something like a diagram of the amount of sugar I consumed based on my last shopping and... a different diagram with the amount of sugar I swapped/reduced by not buying some products and swapping for other options.” |
|  | Personalised app | Participants recommended that the intervention could be personalised to their own unmet physical health needs. | “It would be great if the future app can give suggestions based on consideration of my current health conditions / body information (e.g., height and weight).” |

^a^ Pseudonames are not provided under each quote.

Table S2 – Sensitivity analysis of exploratory effectiveness measures

|  | Baseline | Follow up | Change | Change  adjusted^a^ |  | Between group difference^a^, intervention vs control | |
| --- | --- | --- | --- | --- | --- | --- | --- |
|  | Mean (SD) | Mean (SD) | Mean (95%CI) | Mean (95%CI) |  | Mean (95%CI) | P value |
| *Purchased Sugar (g/100g)* |  |  |  |  |  |  |  |
| Sugar group (n=34) | 5.13 (2.63) | 4.13 (2.34) | -1.00 (-1.97, -0.03) | -0.83 (-1.71, 0.04) |  | -0.58 (-2.15, 0.98)) | 0.454 |
| Control (n=10) | 4.26 (1.49) | 4.58 (1.86) | 0.32 (-1.47, 2.11) | -0.25 (-1.41, 0.91)) |  |  |  |
| *Purchased SFA (g/100g)* |  |  |  |  |  |  |  |
| SFA group (n=28) | 2.13 (1.18) | 1.58 (0.98) | -0.56 (-1.02, -0.10) | -0.60 (-1.05, -0.16) |  | -1.22 (-2.22, -0.21) | 0.019 |
| Control (n=12) | 2.10 (0.84) | 2.61 (1.22) | 0.52 (-0.19, 1.22) | 0.61 (-0.17, 1.40)) |  |  |  |

^a^ Linear regression adjusted for baseline values of the nutrient, plus age, sex, ethnicity, and income


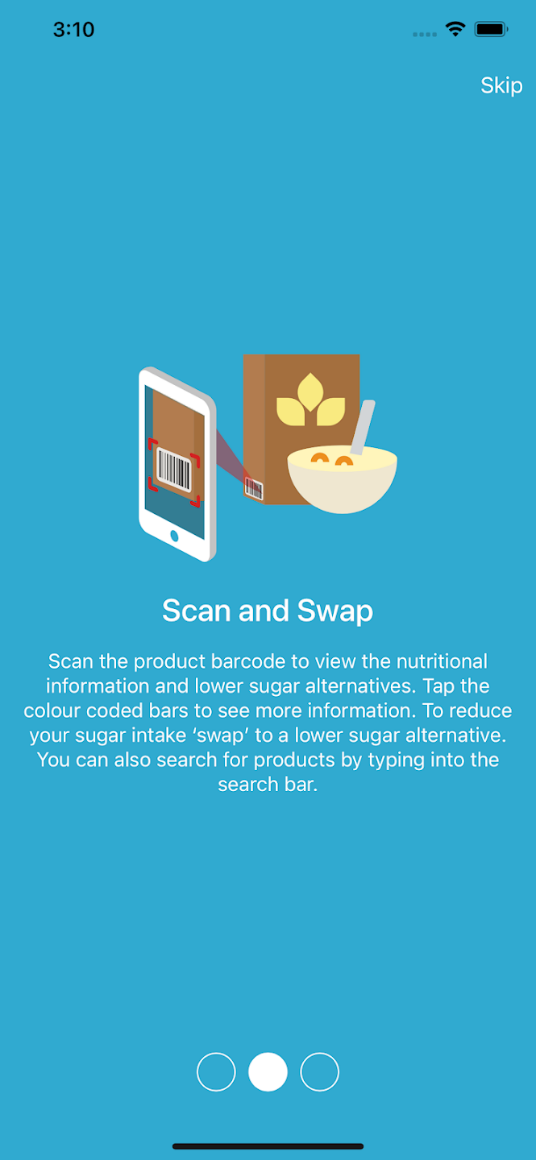
Figure S1. Screenshots of the SwapSHOP app functionality


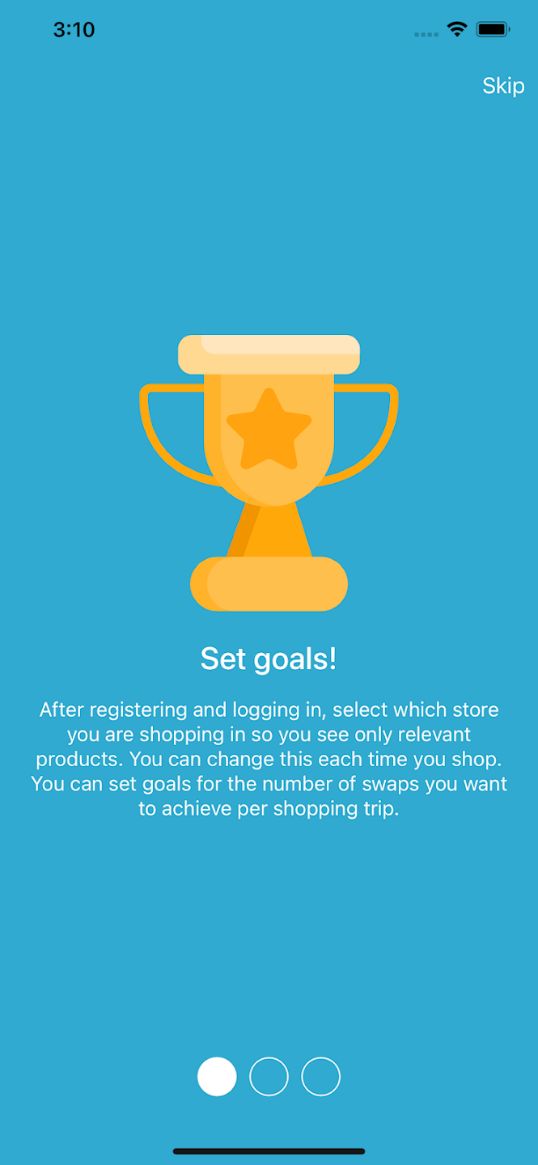

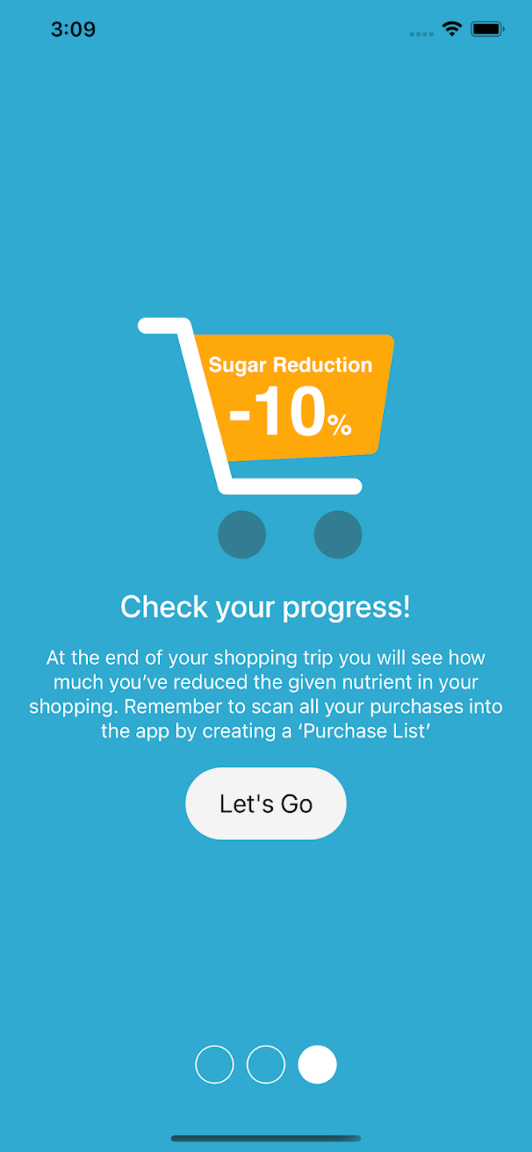


Figure S1. Continuation


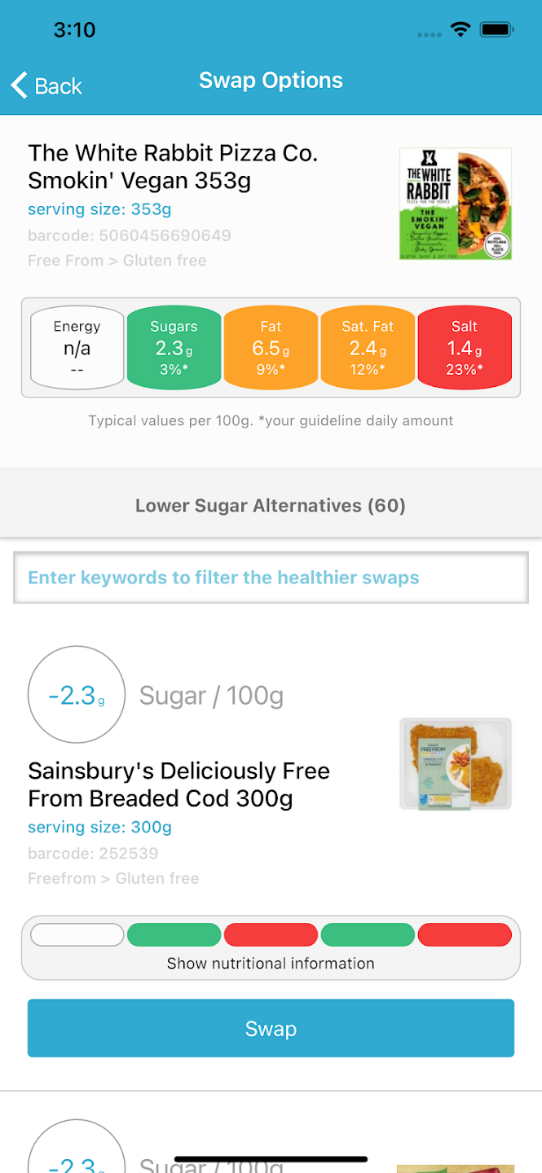

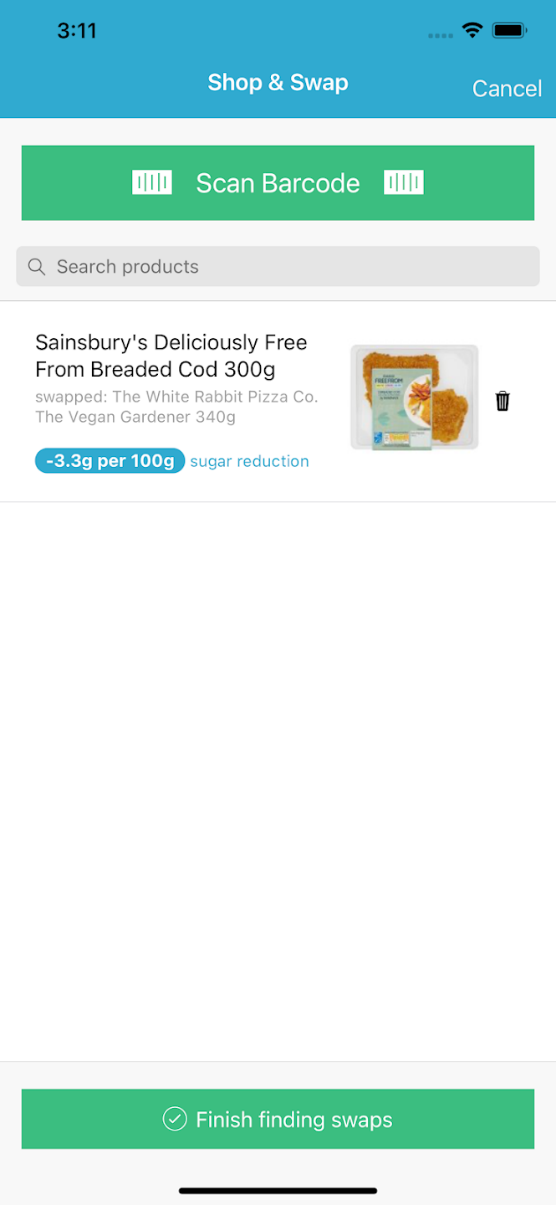

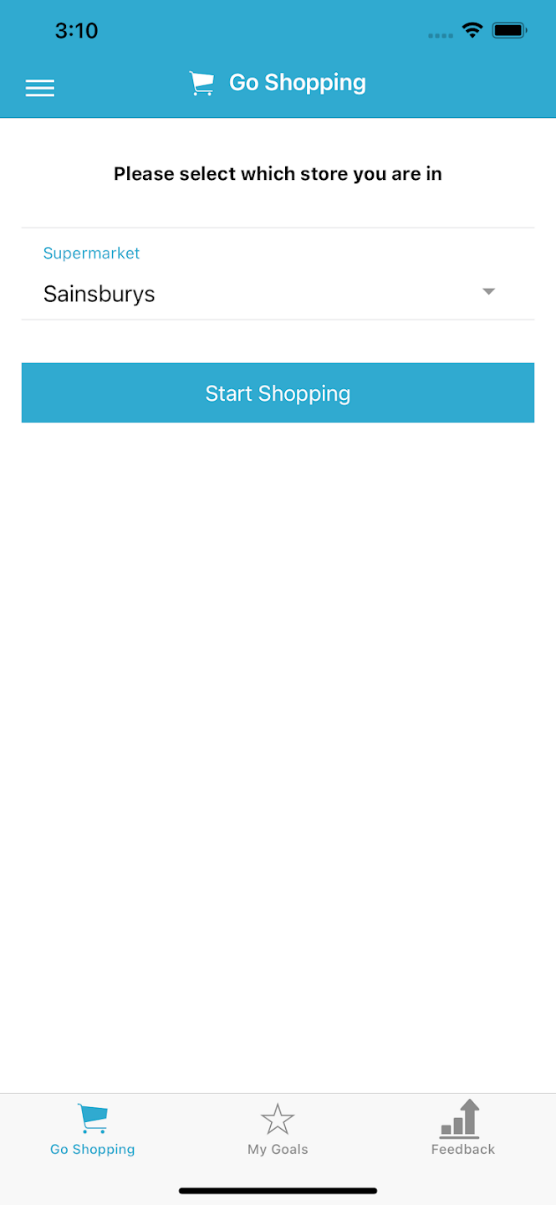
-

.
