# Supplementary material for: A Behaviorally Informed Mobile App to Improve the Nutritional Quality of Grocery Shopping (SwapSHOP): Feasibility Randomized Controlled Trial
Source: JMIR Mhealth Uhealth. 2024 Jan 11;12:e45854. doi: 10.2196/45854 (PMC10811579; doi:10.2196/45854)
Supplement: Multimedia Appendix 2 [file mhealth_v12i1e45854_app2.pdf]

CONSORT-EHEALTH (V 1.6.1) - Submission/Publication Form

The CONSORT-EHEALTH checklist is intended for authors of randomized trials evaluating web-based and Internet-based applications/interventions, including mobile interventions, electronic games (incl multiplayer games), social media, certain telehealth applications, and other interactive and/or networked electronic applications. Some of the items (e.g. all subitems under item 5 - description of the intervention) may also be applicable for other study designs.

The goal of the CONSORT EHEALTH checklist and guideline is to be
a) a guide for reporting for authors of RCTs,
b) to form a basis for appraisal of an ehealth trial (in terms of validity)

CONSORT-EHEALTH items/subitems are MANDATORY reporting items for studies published in the Journal of Medical Internet Research and other journals / scientific societies endorsing the checklist.

Items numbered 1., 2., 3., 4a., 4b etc are original CONSORT or CONSORT-NPT (non-pharmacologic treatment) items.
Items with Roman numerals (i., ii, iii, iv etc.) are CONSORT-EHEALTH extensions/clarifications.

As the CONSORT-EHEALTH checklist is still considered in a formative stage, we would ask that you also RATE ON A SCALE OF 1-5 how important/useful you feel each item is FOR THE PURPOSE OF THE CHECKLIST and reporting guideline (optional).

Mandatory reporting items are marked with a red *.
In the textboxes, either copy & paste the relevant sections from your manuscript into this form - please include any quotes from your manuscript in QUOTATION MARKS,
or answer directly by providing additional information not in the manuscript, or elaborating on why the item was not relevant for this study.

YOUR ANSWERS WILL BE PUBLISHED AS A SUPPLEMENTARY FILE TO YOUR PUBLICATION IN JMIR AND ARE CONSIDERED PART OF YOUR PUBLICATION (IF ACCEPTED).
Please fill in these questions diligently. Information will not be copyedited, so please use proper spelling and grammar, use correct capitalization, and avoid abbreviations.

DO NOT FORGET TO SAVE AS PDF _AND_ CLICK THE SUBMIT BUTTON SO YOUR ANSWERS ARE IN OUR DATABASE !!!

Citation Suggestion (if you append the pdf as Appendix we suggest to cite this paper in the caption):
Eysenbach G, CONSORT-EHEALTH Group
CONSORT-EHEALTH: Improving and Standardizing Evaluation Reports of Web-based and Mobile Health Interventions
J Med Internet Res 2011;13(4):e126

URL: http://www.jmir.org/2011/4/e126/
doi: 10.2196/jmir.1923
[illegible]ID: 22209829

carmenpiernas@gmail.com (not shared) Switch account

Draft saved

* Required

Your name *

First Last

Carmen Piernas

Primary Affiliation (short), City, Country *

University of Toronto, Toronto, Canada

University of Oxford, Oxford UK

Your e-mail address *

abc@gmail.com

carmen.piernas-sanchez@phc.ox.ac.uk

Title of your manuscript *

Provide the (draft) title of your manuscript.

SwapSHOP, a behaviourally-informed mobile app to improve the nutritional quality of the grocery shopping: results of a feasibility randomised controlled trial

Name of your App/Software/Intervention *

If there is a short and a long/alternate name, write the short name first and add the long name in brackets.

SwapSHOP App

Evaluated Version (if any)

e.g. "V1", "Release 2017-03-01", "Version 2.0.27913"

V1 Release 2021-04

Language(s) *

What language is the intervention/app in? If multiple languages are available, separate by comma (e.g. "English, French")

English

URL of your Intervention Website or App

e.g. a direct link to the mobile app on app in appstore (itunes, Google Play), or URL of the website. If the intervention is a DVD or hardware, you can also link to an Amazon page.

Your answer

URL of an image/screenshot (optional)

N/A

Accessibility *

Can an enduser access the intervention presently?

- ( ) access is free and open
- ( ) access only for special usergroups, not open
- ( ) access is open to everyone, but requires payment/subscription/in-app purchases
- (x) app/intervention no longer accessible
- ( ) Other:

Primary Medical Indication/Disease/Condition *

e.g. "Stress", "Diabetes", or define the target group in brackets after the condition, e.g. "Autism (Parents of children with)", "Alzheimers (Informal Caregivers of)"

chronic disease prevention

Primary Outcomes measured in trial *

comma-separated list of primary outcomes reported in the trial

The primary objective of this study was to dete

Secondary/other outcomes

Are there any other outcomes the intervention is expected to affect?

Secondary outcomes
Feasibility outcomes included: 1) Recruitment rates: total recruited (including number signed up, eligible, consented, and randomised); 2) Period of time needed for recruitment of the final sample; 3) Outcome reporting: number of participants who fail to scan their purchases for a minimum of 2 weeks during follow up; number of participants who fail to complete the end-of-study questionnaires.
Process evaluation and qualitative outcome measures
A summary of app-related usage (within-app automatic recording) and acceptability measures collected through the end-of-study questionnaires at follow up: 1) Average number of shopping trips where the app was used to scan products in order to obtain a swap per week; 2) Number of occasions the app was used to scan products for a swap per trip; 3) Number of swaps made overall per week and per shopping trip; 4) Nutrient (SFA, sugar, and salt) reduction per swap made; 5) Use of specific functionality e.g. goal setting, feedback; 6) End-of-study questionnaires with free text to understand app usage and acceptability of the swaps; app functionality (e.g. if app scans the majority of products) and if this prompted other behaviours such as reading nutrition labels.

Exploratory effectiveness outcomes
Measures included changes in the nutrient content (SFA and sugar in g per 100 g) of household food purchases recorded in the app, between baseline and follow up in the intervention group compared to control.

Recommended "Dose" *

What do the instructions for users say on how often the app should be used?

- (●) Approximately Daily
- ( ) Approximately Weekly
- ( ) Approximately Monthly
- ( ) Approximately Yearly
- ( ) "as needed"
- ( ) Other:

Approx. Percentage of Users (starters) still using the app as recommended after 3 months *

- (selected) unknown / not evaluated
- 0-10%
- 11-20%
- 21-30%
- 31-40%
- 41-50%
- 51-60%
- 61-70%
- 71%-80%
- 81-90%
- 91-100%
- Other:

Overall, was the app/intervention effective? *

- (selected) yes: all primary outcomes were significantly better in intervention group vs control
- partly: SOME primary outcomes were significantly better in intervention group vs control
- no statistically significant difference between control and intervention
- potentially harmful: control was significantly better than intervention in one or more outcomes
- inconclusive: more research is needed
- Other:

Article Preparation Status/Stage *

At which stage in your article preparation are you currently (at the time you fill in this form)

- ○ not submitted yet - in early draft status
- ○ not submitted yet - in late draft status, just before submission
- ◉ submitted to a journal but not reviewed yet
- ○ submitted to a journal and after receiving initial reviewer comments
- ○ submitted to a journal and accepted, but not published yet
- ○ published
- ○ Other:

Journal *

If you already know where you will submit this paper (or if it is already submitted), please provide the journal name (if it is not JMIR, provide the journal name under "other")

- ○ not submitted yet / unclear where I will submit this
- ◉ Journal of Medical Internet Research (JMIR)
- ○ JMIR mHealth and UHealth
- ○ JMIR Serious Games
- ○ JMIR Mental Health
- ○ JMIR Public Health
- ○ JMIR Formative Research
- ○ Other JMIR sister journal
- ○ Other:

Is this a full powered effectiveness trial or a pilot/feasibility trial? *

- (x) Pilot/feasibility
- ( ) Fully powered

Manuscript tracking number *

If this is a JMIR submission, please provide the manuscript tracking number under "other" (The ms tracking number can be found in the submission acknowledgement email, or when you login as author in JMIR. If the paper is already published in JMIR, then the ms tracking number is the four-digit number at the end of the DOI, to be found at the bottom of each published article in JMIR)

- (x) no ms number (yet) / not (yet) submitted to / published in JMIR
- ( ) Other:

TITLE AND ABSTRACT

1a) TITLE: Identification as a randomized trial in the title

1a) Does your paper address CONSORT item 1a? *

I.e does the title contain the phrase "Randomized Controlled Trial"? (if not, explain the reason under "other")

- (x) yes
- ( ) Other:

1a-i) Identify the mode of delivery in the title

Identify the mode of delivery. Preferably use “web-based” and/or “mobile” and/or “electronic game” in the title. Avoid ambiguous terms like “online”, “virtual”, “interactive”. Use “Internet-based” only if Intervention includes non-web-based Internet components (e.g. email), use “computer-based” or “electronic” only if offline products are used. Use “virtual” only in the context of “virtual reality” (3-D worlds). Use “online” only in the context of “online support groups”. Complement or substitute product names with broader terms for the class of products (such as “mobile” or “smart phone” instead of “iphone”), especially if the application runs on different platforms.

subitem not at all important

1 ○

2 ○

3 ○

4 ○

5 ◉

essential

Clear selection

Does your paper address subitem 1a-i? *

Copy and paste relevant sections from manuscript title (include quotes in quotation marks "like this" to indicate direct quotes from your manuscript), or elaborate on this item by providing additional information not in the ms, or briefly explain why the item is not applicable/relevant for your study

This study aimed to assess the feasibility and acceptability of receiving advice on healthier food purchases through SwapSHOP, a behaviourally-informed smartphone app that allows users to scan barcodes of grocery products from the United Kingdom, providing nutritional information and personalised swap suggestions to encourage healthier purchases.

1a-ii) Non-web-based components or important co-interventions in title

Mention non-web-based components or important co-interventions in title, if any (e.g., “with telephone support”).

subitem not at all important

1 (selected)

2

3

4

5

essential

Clear selection

Does your paper address subitem 1a-ii?

Copy and paste relevant sections from manuscript title (include quotes in quotation marks "like this" to indicate direct quotes from your manuscript), or elaborate on this item by providing additional information not in the ms, or briefly explain why the item is not applicable/relevant for your study

Your answer

1a-iii) Primary condition or target group in the title

Mention primary condition or target group in the title, if any (e.g., “for children with Type I Diabetes”) Example: A Web-based and Mobile Intervention with Telephone Support for Children with Type I Diabetes: Randomized Controlled Trial

subitem not at all important

- 1 (selected)
- 2
- 3
- 4
- 5

essential

Clear selection

Does your paper address subitem 1a-iii? *

Copy and paste relevant sections from manuscript title (include quotes in quotation marks "like this" to indicate direct quotes from your manuscript), or elaborate on this item by providing additional information not in the ms, or briefly explain why the item is not applicable/relevant for your study

SwapSHOP, a behaviourally-informed mobile app to improve the nutritional quality of the grocery shopping

1b) ABSTRACT: Structured summary of trial design, methods, results, and conclusions

NPT extension: Description of experimental treatment, comparator, care providers, centers, and blinding status.

1b-i) Key features/functionalities/components of the intervention and comparator in the METHODS section of the ABSTRACT

Mention key features/functionalities/components of the intervention and comparator in the abstract. If possible, also mention theories and principles used for designing the site. Keep in mind the needs of systematic reviewers and indexers by including important synonyms. (Note: Only report in the abstract what the main paper is reporting. If this information is missing from the main body of text, consider adding it)

subitem not at all important

1 ○

2 ○

3 ○

4 ○

5 ◉

essential

Clear selection

Does your paper address subitem 1b-i? *

Copy and paste relevant sections from the manuscript abstract (include quotes in quotation marks "like this" to indicate direct quotes from your manuscript), or elaborate on this item by providing additional information not in the ms, or briefly explain why the item is not applicable/relevant for your study

We randomised adult volunteers in a 6-arm parallel-group controlled feasibility trial. Participants used the SwapSHOP app to record their grocery shopping during a 2-week run-in period and were individually randomised 3:1 to either intervention or control arms within three strata related to a nutrient of concern of their choice - saturated fat (SFA), sugar or salt. Participants randomised to the intervention received the SwapSHOP app with a healthier swap function, goal-setting and personalised feedback. The control group were asked to use the simpler version of the app to record all their food purchases with no advice. The primary outcome assessed the feasibility of progression to a full trial, including app use and follow up rates at 6-weeks. Secondary outcomes included other feasibility outcomes, process and qualitative measures and exploratory effectiveness outcomes to assess changes in the nutrient content of purchased foods.

1b-ii) Level of human involvement in the METHODS section of the ABSTRACT

Clarify the level of human involvement in the abstract, e.g., use phrases like “fully automated” vs. “therapist/nurse/care provider/physician-assisted” (mention number and expertise of providers involved, if any). (Note: Only report in the abstract what the main paper is reporting. If this information is missing from the main body of text, consider adding it)

subitem not at all important

- 1 (selected)
- 2
- 3
- 4
- 5

essential

Clear selection

Does your paper address subitem 1b-ii?

Copy and paste relevant sections from the manuscript abstract (include quotes in quotation marks "like this" to indicate direct quotes from your manuscript), or elaborate on this item by providing additional information not in the ms, or briefly explain why the item is not applicable/relevant for your study

Your answer

1b-iii) Open vs. closed, web-based (self-assessment) vs. face-to-face assessments in the METHODS section of the ABSTRACT

Mention how participants were recruited (online vs. offline), e.g., from an open access website or from a clinic or a closed online user group (closed usergroup trial), and clarify if this was a purely web-based trial, or there were face-to-face components (as part of the intervention or for assessment). Clearly say if outcomes were self-assessed through questionnaires (as common in web-based trials). Note: In traditional offline trials, an open trial (open-label trial) is a type of clinical trial in which both the researchers and participants know which treatment is being administered. To avoid confusion, use “blinded” or “unblinded” to indicated the level of blinding instead of “open”, as “open” in web-based trials usually refers to “open access” (i.e. participants can self-enrol). (Note: Only report in the abstract what the main paper is reporting. If this information is missing from the main body of text, consider adding it)

subitem not at all important

- 1 (selected)
- 2
- 3
- 4
- 5

essential

Clear selection

Does your paper address subitem 1b-iii?

Copy and paste relevant sections from the manuscript abstract (include quotes in quotation marks "like this" to indicate direct quotes from your manuscript), or elaborate on this item by providing additional information not in the ms, or briefly explain why the item is not applicable/relevant for your study

Your answer

1b-iv) RESULTS section in abstract must contain use data

Report number of participants enrolled/assessed in each group, the use/uptake of the intervention (e.g., attrition/adherence metrics, use over time, number of logins etc.), in addition to primary/secondary outcomes. (Note: Only report in the abstract what the main paper is reporting. If this information is missing from the main body of text, consider adding it)

subitem not at all important

1 ○

2 ○

3 ○

4 ○

5 ◉

essential

Clear selection

Does your paper address subitem 1b-iv?

Copy and paste relevant sections from the manuscript abstract (include quotes in quotation marks "like this" to indicate direct quotes from your manuscript), or elaborate on this item by providing additional information not in the ms, or briefly explain why the item is not applicable/relevant for your study

The two progression criteria were met for SFA and sugar: 81% and 87% of intervention participants in the SFA and sugar group respectively used the app to obtain healthier swaps; and 96% of intervention participants and 89% of control participants completed follow-up by scanning all purchases over the follow-up period.

1b-v) CONCLUSIONS/DISCUSSION in abstract for negative trials

Conclusions/Discussions in abstract for negative trials: Discuss the primary outcome - if the trial is negative (primary outcome not changed), and the intervention was not used, discuss whether negative results are attributable to lack of uptake and discuss reasons. (Note: Only report in the abstract what the main paper is reporting. If this information is missing from the main body of text, consider adding it)

subitem not at all important

- 1 (selected)
- 2
- 3
- 4
- 5

essential

Clear selection

Does your paper address subitem 1b-v?

Copy and paste relevant sections from the manuscript abstract (include quotes in quotation marks "like this" to indicate direct quotes from your manuscript), or elaborate on this item by providing additional information not in the ms, or briefly explain why the item is not applicable/relevant for your study

Your answer

INTRODUCTION

2a) In INTRODUCTION: Scientific background and explanation of rationale

2a-i) Problem and the type of system/solution

Describe the problem and the type of system/solution that is object of the study: intended as stand-alone intervention vs. incorporated in broader health care program? Intended for a particular patient population? Goals of the intervention, e.g., being more cost-effective to other interventions, replace or complement other solutions? (Note: Details about the intervention are provided in "Methods" under 5)

subitem not at all important

1 ○

2 ○

3 ○

4 ○

5 ◉

essential

Clear selection

Does your paper address subitem 2a-i? *

Copy and paste relevant sections from the manuscript (include quotes in quotation marks "like this" to indicate direct quotes from your manuscript), or elaborate on this item by providing additional information not in the ms, or briefly explain why the item is not applicable/relevant for your study

Consumption of saturated fat (SFA), sugars and salt in the United Kingdom (UK) and most high-income countries currently exceeds dietary recommendations for good health (1). These nutrients of concern contribute to the burden of diabetes and cardiovascular disease (CVD), either directly or through effects on blood cholesterol, blood pressure, insulin sensitivity and body weight (2, 3, 4, 5, 6, 7, 8). Despite decades of nutrition education and efforts to promote healthier behaviours, significant and sustained dietary change is yet to be seen. Evidence suggests people need more than general knowledge of dietary recommendations to change behaviour (9, 10, 11), however there is limited evidence for individual-level interventions that are feasible and effective to support dietary change at a population-level.
Food purchasing is a key antecedent of food consumption and improving the nutritional quality of food purchases presents a clear opportunity to intervene. Grocery stores account for 71% of the weekly expenditure on food and drinks, including a substantial proportion of foods high in saturated fat (SFA), sugar and salt (12). For many foods, there are usually alternatives with less SFA, sugar or salt, which are functionally similar. This is partly due to natural variation in recipes e.g. ready meals or cost of ingredients, but also specific reformulation of products in response to consumer demand e.g. low fat yoghurts, or policy actions e.g. soft drinks(13).

2a-ii) Scientific background, rationale: What is known about the (type of) system

Scientific background, rationale: What is known about the (type of) system that is the object of the study (be sure to discuss the use of similar systems for other conditions/diagnoses, if appropiate), motivation for the study, i.e. what are the reasons for and what is the context for this specific study, from which stakeholder viewpoint is the study performed, potential impact of findings [2]. Briefly justify the choice of the comparator.

subitem not at all important

1 ○

2 ○

3 ○

4 ○

5 ◉

essential

Clear selection

Does your paper address subitem 2a-ii? *

Copy and paste relevant sections from the manuscript (include quotes in quotation marks "like this" to indicate direct quotes from your manuscript), or elaborate on this item by providing additional information not in the ms, or briefly explain why the item is not applicable/relevant for your study

Systematic reviews have identified some intervention components to support individual dietary change, including providing information, tailored dietary advice, self-monitoring and personalised feedback (9, 14, 15). Specifically recommending healthier swaps, at the point of purchase or as part of individually-tailored regular feedback, has shown potential to help improve the nutritional quality of the grocery shopping (16, 17, 18, 19, 20). Technological advances such as smartphone apps can facilitate this, providing scalable, lower-cost support to help people make healthier choices while shopping.
A systematic review of smartphone apps only identified two studies testing apps of this kind (21). These apps mostly provide healthier swaps to consumers without the option to self-monitor the quality of their grocery shopping, and many have limitations regarding how information is presented to consumers, or the limited comprehensiveness of the databases covering the UK food market (22, 23). Our recent proof-of-concept study tested the functionality of the SaltSwap app to help find lower-salt foods when grocery shopping, which included behavioural components such as nutritional information, prompts to lower salt options, goal setting on swaps, feedback on swaps and salt reduction, and history of swaps (20).

2b) In INTRODUCTION: Specific objectives or hypotheses

Does your paper address CONSORT subitem 2b? *

Copy and paste relevant sections from the manuscript (include quotes in quotation marks "like this" to indicate direct quotes from your manuscript), or elaborate on this item by providing additional information not in the ms, or briefly explain why the item is not applicable/relevant for your study

Here we have further developed the SaltSwap app into SwapSHOP, a new app that also provide swaps for SFA and sugars as well as salt. This study aimed to assess the feasibility and acceptability of receiving dietary advice through the SwapSHOP app. It as a standalone intervention that allowed users to scan barcodes of grocery products from major UK stores to obtain nutritional information and suggestions for personalised swaps with lower SFA, sugar or salt and also enabled users to set goals and monitor their shopping behaviour.

METHODS

3a) Description of trial design (such as parallel, factorial) including allocation ratio

Does your paper address CONSORT subitem 3a? *

Copy and paste relevant sections from the manuscript (include quotes in quotation marks "like this" to indicate direct quotes from your manuscript), or elaborate on this item by providing additional information not in the ms, or briefly explain why the item is not applicable/relevant for your study

This was a prospectively registered (ISRCTN13022312), randomised, parallel 6-arm controlled trial conducted in the UK. After giving written informed consent to participate in the study and completing screening and baseline assessment, participants entered a 2-week run-in period where they used a basic version of the SwapSHOP app (no swap/behavioural functionality shown) to record their grocery shopping. Only participants who completed this task with good engagement with the app were randomised. Good engagement was defined as scanning products during at least two shops, each with products from at least three different pre-defined food categories (e.g. fresh meat, chilled ready meals, soft drinks, etc.), and scanning products from five or more of the food categories across the two weeks. They were individually randomised to one of the intervention arms or control following a 3:1 ratio (intervention: control) within three strata related to a nutrient of concern of their choice (SFA, sugar or salt). Individuals participated in the study over 6 weeks from screening to final follow-up.

3b) Important changes to methods after trial commencement (such as eligibility criteria), with reasons

Does your paper address CONSORT subitem 3b? *

Copy and paste relevant sections from the manuscript (include quotes in quotation marks "like this" to indicate direct quotes from your manuscript), or elaborate on this item by providing additional information not in the ms, or briefly explain why the item is not applicable/relevant for your study

No changes after trial commencement

3b-i) Bug fixes, Downtimes, Content Changes

Bug fixes, Downtimes, Content Changes: ehealth systems are often dynamic systems. A description of changes to methods therefore also includes important changes made on the intervention or comparator during the trial (e.g., major bug fixes or changes in the functionality or content) (5-iii) and other “unexpected events” that may have influenced study design such as staff changes, system failures/downtimes, etc. [2].

subitem not at all important

- 1 (selected)
- 2
- 3
- 4
- 5

essential

Clear selection

Does your paper address subitem 3b-i?

Copy and paste relevant sections from the manuscript (include quotes in quotation marks "like this" to indicate direct quotes from your manuscript), or elaborate on this item by providing additional information not in the ms, or briefly explain why the item is not applicable/relevant for your study

Your answer

4a) Eligibility criteria for participants

Does your paper address CONSORT subitem 4a? *

Copy and paste relevant sections from the manuscript (include quotes in quotation marks "like this" to indicate direct quotes from your manuscript), or elaborate on this item by providing additional information not in the ms, or briefly explain why the item is not applicable/relevant for your study

Participants were eligible if they were over the age of 18, willing and able to give informed consent, English speaking and able to understand the demands of the study, looking for support to improve their diet, owning a smartphone with access to the internet and email account, willing to download and use a smartphone app to scan and track their grocery shopping for the 6 weeks during their participation in the study, and responsible for at least some of their household grocery shopping, regularly shopping (e.g. at least once a week in a physical store or online in one of six major food retailers in the UK (Tesco, Sainsbury's, Waitrose, Asda, Morrisons, or Iceland). People were not eligible if they were already on a clinician-supervised diet or a restricted diet, were currently using apps to monitor the quality of their food shopping (excluding apps to track and monitor food intake), were currently participating in another study aimed at dietary change or asking them to change the way they shop for food, or were planning on going away from home (holiday or other) for more than 2 consecutive weeks following

4a-i) Computer / Internet literacy

Computer / Internet literacy is often an implicit "de facto" eligibility criterion - this should be explicitly clarified.

subitem not at all important

1 ○

2 ○

3 ○

4 ○

5 ◉

essential

Clear selection

Does your paper address subitem 4a-i?

Copy and paste relevant sections from the manuscript (include quotes in quotation marks "like this" to indicate direct quotes from your manuscript), or elaborate on this item by providing additional information not in the ms, or briefly explain why the item is not applicable/relevant for your study

owning a smartphone with access to the internet and email account, willing to download and use a smartphone app to scan and track their grocery shopping for the 6 weeks during their participation in the study,

4a-ii) Open vs. closed, web-based vs. face-to-face assessments:

Open vs. closed, web-based vs. face-to-face assessments: Mention how participants were recruited (online vs. offline), e.g., from an open access website or from a clinic, and clarify if this was a purely web-based trial, or there were face-to-face components (as part of the intervention or for assessment), i.e., to what degree got the study team to know the participant. In online-only trials, clarify if participants were quasi-anonymous and whether having multiple identities was possible or whether technical or logistical measures (e.g., cookies, email confirmation, phone calls) were used to detect/prevent these.

subitem not at all important

1 ○

2 ○

3 ○

4 ○

5 ◉

essential

Clear selection

Does your paper address subitem 4a-ii? *

Copy and paste relevant sections from the manuscript (include quotes in quotation marks "like this" to indicate direct quotes from your manuscript), or elaborate on this item by providing additional information not in the ms, or briefly explain why the item is not applicable/relevant for your study

Participants were recruited between April and October 2021 through the community (e.g., word-of-mouth) and through online social networks and media, online newsletters and newspapers, and electronic mailing lists. Eligible participants were invited to complete a baseline online questionnaire where demographic information, relevant self-reported medical history and shopping behaviours were collected. Participants also reported which nutrient (SFA, sugar or salt) they were most concerned about in their diet, which was used as stratification factor during the randomisation process. Participants then entered a 2-week run-in period during which they were asked to record all their food purchases, either by scanning the barcode of purchased grocery products or manually inputting what was purchased using a simpler version of the SwapSHOP app.

4a-iii) Information giving during recruitment

Information given during recruitment. Specify how participants were briefed for recruitment and in the informed consent procedures (e.g., publish the informed consent documentation as appendix, see also item X26), as this information may have an effect on user self-selection, user expectation and may also bias results.

subitem not at all important

- 1 (selected)
- 2
- 3
- 4
- 5

essential

Clear selection

Does your paper address subitem 4a-iii?

Copy and paste relevant sections from the manuscript (include quotes in quotation marks "like this" to indicate direct quotes from your manuscript), or elaborate on this item by providing additional information not in the ms, or briefly explain why the item is not applicable/relevant for your study

Your answer

4b) Settings and locations where the data were collected

Does your paper address CONSORT subitem 4b? *

Copy and paste relevant sections from the manuscript (include quotes in quotation marks "like this" to indicate direct quotes from your manuscript), or elaborate on this item by providing additional information not in the ms, or briefly explain why the item is not applicable/relevant for your study

Eligible participants were invited to complete a baseline online questionnaire where demographic information, relevant self-reported medical history and shopping behaviours were collected. Participants also reported which nutrient (SFA, sugar or salt) they were most concerned about in their diet, which was used as stratification factor during the randomisation process. Participants then entered a 2-week run-in period during which they were asked to record all their food purchases, either by scanning the barcode of purchased grocery products or manually inputting what was purchased using a simpler version of the SwapSHOP app.

4b-i) Report if outcomes were (self-)assessed through online questionnaires

Clearly report if outcomes were (self-)assessed through online questionnaires (as common in web-based trials) or otherwise.

subitem not at all important

1 ○

2 ○

3 ○

4 ○

5 ◉

essential

Clear selection

Does your paper address subitem 4b-i? *

Copy and paste relevant sections from the manuscript (include quotes in quotation marks "like this" to indicate direct quotes from your manuscript), or elaborate on this item by providing additional information not in the ms, or briefly explain why the item is not applicable/relevant for your study

The primary objective of this study was to determine the feasibility of a larger study to evaluate the effectiveness of an app that offers healthier swaps while grocery shopping to improve the nutritional quality of food purchasing with respect to sugar, salt or saturated fat. The primary outcomes were pre-specified progression criteria as follows:
1) App use: at least 70% of participants in the active intervention groups use the app to obtain swaps on at least one occasion by the end of the second week after randomisation.
2) Follow up rate: at least 60% of participants in total (intervention and control) complete follow-up by scanning all their purchases for a minimum of 2 weeks over the entire follow up period (4 weeks).

4b-ii) Report how institutional affiliations are displayed

Report how institutional affiliations are displayed to potential participants [on ehealth media], as affiliations with prestigious hospitals or universities may affect volunteer rates, use, and reactions with regards to an intervention.(Not a required item – describe only if this may bias results)

subitem not at all important

1 (selected)

2

3

4

5

essential

Clear selection

Does your paper address subitem 4b-ii?

Copy and paste relevant sections from the manuscript (include quotes in quotation marks "like this" to indicate direct quotes from your manuscript), or elaborate on this item by providing additional information not in the ms, or briefly explain why the item is not applicable/relevant for your study

Your answer

5) The interventions for each group with sufficient details to allow replication, including how and when they were actually administered

5-i) Mention names, credential, affiliations of the developers, sponsors, and owners

Mention names, credential, affiliations of the developers, sponsors, and owners [6] (if authors/evaluators are owners or developer of the software, this needs to be declared in a “Conflict of interest” section or mentioned elsewhere in the manuscript).

subitem not at all important

1 (selected)

2

3

4

5

essential

Clear selection

Does your paper address subitem 5-i?

Copy and paste relevant sections from the manuscript (include quotes in quotation marks "like this" to indicate direct quotes from your manuscript), or elaborate on this item by providing additional information not in the ms, or briefly explain why the item is not applicable/relevant for your study

Your answer

5-ii) Describe the history/development process

Describe the history/development process of the application and previous formative evaluations (e.g., focus groups, usability testing), as these will have an impact on adoption/use rates and help with interpreting results.

subitem not at all important

1 (selected)

2

3

4

5

essential

Clear selection

Does your paper address subitem 5-ii?

Copy and paste relevant sections from the manuscript (include quotes in quotation marks "like this" to indicate direct quotes from your manuscript), or elaborate on this item by providing additional information not in the ms, or briefly explain why the item is not applicable/relevant for your study

Your answer

5-iii) Revisions and updating

Revisions and updating. Clearly mention the date and/or version number of the application/intervention (and comparator, if applicable) evaluated, or describe whether the intervention underwent major changes during the evaluation process, or whether the development and/or content was “frozen” during the trial. Describe dynamic components such as news feeds or changing content which may have an impact on the replicability of the intervention (for unexpected events see item 3b).

subitem not at all important

- 1 (selected)
- 2
- 3
- 4
- 5

essential

Clear selection

Does your paper address subitem 5-iii?

Copy and paste relevant sections from the manuscript (include quotes in quotation marks "like this" to indicate direct quotes from your manuscript), or elaborate on this item by providing additional information not in the ms, or briefly explain why the item is not applicable/relevant for your study

Your answer

5-iv) Quality assurance methods

Provide information on quality assurance methods to ensure accuracy and quality of information provided [1], if applicable.

subitem not at all important

1 (selected)

2

3

4

5

essential

Clear selection

Does your paper address subitem 5-iv?

Copy and paste relevant sections from the manuscript (include quotes in quotation marks "like this" to indicate direct quotes from your manuscript), or elaborate on this item by providing additional information not in the ms, or briefly explain why the item is not applicable/relevant for your study

Your answer

5-v) Ensure replicability by publishing the source code, and/or providing screenshots/screen-capture video, and/or providing flowcharts of the algorithms used

Ensure replicability by publishing the source code, and/or providing screenshots/screen-capture video, and/or providing flowcharts of the algorithms used. Replicability (i.e., other researchers should in principle be able to replicate the study) is a hallmark of scientific reporting.

subitem not at all important

- 1 (selected)
- 2
- 3
- 4
- 5

essential

Clear selection

Does your paper address subitem 5-v?

Copy and paste relevant sections from the manuscript (include quotes in quotation marks "like this" to indicate direct quotes from your manuscript), or elaborate on this item by providing additional information not in the ms, or briefly explain why the item is not applicable/relevant for your study

Your answer

5-vi) Digital preservation

Digital preservation: Provide the URL of the application, but as the intervention is likely to change or disappear over the course of the years; also make sure the intervention is archived (Internet Archive, webcitation.org, and/or publishing the source code or screenshots/videos alongside the article). As pages behind login screens cannot be archived, consider creating demo pages which are accessible without login.

subitem not at all important

1 (selected)

2

3

4

5

essential

Clear selection

Does your paper address subitem 5-vi?

Copy and paste relevant sections from the manuscript (include quotes in quotation marks "like this" to indicate direct quotes from your manuscript), or elaborate on this item by providing additional information not in the ms, or briefly explain why the item is not applicable/relevant for your study

Your answer

5-vii) Access

Access: Describe how participants accessed the application, in what setting/context, if they had to pay (or were paid) or not, whether they had to be a member of specific group. If known, describe how participants obtained "access to the platform and Internet" [1]. To ensure access for editors/reviewers/readers, consider to provide a "backdoor" login account or demo mode for reviewers/readers to explore the application (also important for archiving purposes, see vi).

subitem not at all important

1 ○

2 ○

3 ◉

4 ○

5 ○

essential

Clear selection

Does your paper address subitem 5-vii? *

Copy and paste relevant sections from the manuscript (include quotes in quotation marks "like this" to indicate direct quotes from your manuscript), or elaborate on this item by providing additional information not in the ms, or briefly explain why the item is not applicable/relevant for your study

After giving written informed consent to participate in the study and completing screening and baseline assessment, participants entered a 2-week run-in period where they used a basic version of the SwapSHOP app (no swap/behavioural functionality shown) to record their grocery shopping. Only participants who completed this task with good engagement with the app were randomised. Good engagement was defined as scanning products during at least two shops, each with products from at least three different pre-defined food categories (e.g. fresh meat, chilled ready meals, soft drinks, etc.), and scanning products from five or more of the food categories across the two weeks

5-viii) Mode of delivery, features/functionalities/components of the intervention and comparator, and the theoretical framework

Describe mode of delivery, features/functionalities/components of the intervention and comparator, and the theoretical framework [6] used to design them (instructional strategy [1], behaviour change techniques, persuasive features, etc., see e.g., [7, 8] for terminology). This includes an in-depth description of the content (including where it is coming from and who developed it) [1]," whether [and how] it is tailored to individual circumstances and allows users to track their progress and receive feedback" [6]. This also includes a description of communication delivery channels and – if computer-mediated communication is a component – whether communication was synchronous or asynchronous [6]. It also includes information on presentation strategies [1], including page design principles, average amount of text on pages, presence of hyperlinks to other resources, etc. [1].

subitem not at all important

- 1 ○
- 2 ○
- 3 ○
- 4 ○
- 5 ◉

essential

Clear selection

Does your paper address subitem 5-viii? *

Copy and paste relevant sections from the manuscript (include quotes in quotation marks "like this" to indicate direct quotes from your manuscript), or elaborate on this item by providing additional information not in the ms, or briefly explain why the item is not applicable/relevant for your study

Intervention
Participants randomised to the intervention arms were able to access an enhanced version of the SwapSHOP app which included a healthier swap function, as well as goal-setting and personalised feedback. Participants scanned the barcode of grocery products to receive nutritional information about the product using the UK traffic light system, and were presented with alternatives which were lower in SFA, sugar or salt (depending on group allocation). Participants could also set goals for the number of swaps they wanted to make in each shopping trip, and record the products they swapped in the app. The app provided feedback on achievement of their goal and feedback on the overall nutrient reduction achieved through making swaps. The app also displayed a ranking of all purchased foods contributing the most to each nutrient of concern.
The SwapSHOP app included a comprehensive database of >70,000 grocery products available in the major UK grocery stores (including Morrisons, Sainsbury's, Tesco, Waitrose, Iceland and Asda)(24) and a bespoke system for categorising products and selecting suitable alternative swap suggestions. SwapSHOP was based on a previous version developed exclusively for salt and its theoretical basis is grounded in the Behaviour Change Wheel(20, 25), a framework that integrates the elements of capability, opportunity and motivation which are key for behaviour change. A range of behaviour change techniques from the taxonomy groups goals and planning and feedback and monitoring that were incorporated into this intervention have been associated with successful dietary change (9, 26).
In order to enable assessment of changes in the nutritional composition of the shopping baskets, participants received weekly reminders to continue scanning and recording all their purchased products for the remaining 4 weeks of follow up (with a minimum of 2 full weeks of grocery shopping data during the follow up period).
Comparator
Participants randomised to the control arm were asked to continue using the simpler version of the app with no product nutrition information, swaps or behavioural components, solely to record all their food purchases as part of the outcome assessment at the end of six-week follow up.

5-ix) Describe use parameters

Describe use parameters (e.g., intended “doses” and optimal timing for use). Clarify what instructions or recommendations were given to the user, e.g., regarding timing, frequency, heaviness of use, if any, or was the intervention used ad libitum.

subitem not at all important

1 (selected)

2

3

4

5

essential

Clear selection

Does your paper address subitem 5-ix?

Copy and paste relevant sections from the manuscript (include quotes in quotation marks "like this" to indicate direct quotes from your manuscript), or elaborate on this item by providing additional information not in the ms, or briefly explain why the item is not applicable/relevant for your study

Your answer

5-x) Clarify the level of human involvement

Clarify the level of human involvement (care providers or health professionals, also technical assistance) in the e-intervention or as co-intervention (detail number and expertise of professionals involved, if any, as well as "type of assistance offered, the timing and frequency of the support, how it is initiated, and the medium by which the assistance is delivered". It may be necessary to distinguish between the level of human involvement required for the trial, and the level of human involvement required for a routine application outside of a RCT setting (discuss under item 21 – generalizability).

subitem not at all important

- 1 (selected)
- 2
- 3
- 4
- 5

essential

Clear selection

Does your paper address subitem 5-x?

Copy and paste relevant sections from the manuscript (include quotes in quotation marks "like this" to indicate direct quotes from your manuscript), or elaborate on this item by providing additional information not in the ms, or briefly explain why the item is not applicable/relevant for your study

Your answer

5-xi) Report any prompts/reminders used

Report any prompts/reminders used: Clarify if there were prompts (letters, emails, phone calls, SMS) to use the application, what triggered them, frequency etc. It may be necessary to distinguish between the level of prompts/reminders required for the trial, and the level of prompts/reminders for a routine application outside of a RCT setting (discuss under item 21 – generalizability).

subitem not at all important

- 1 (selected)
- 2
- 3
- 4
- 5

essential

Clear selection

Does your paper address subitem 5-xi? *

Copy and paste relevant sections from the manuscript (include quotes in quotation marks "like this" to indicate direct quotes from your manuscript), or elaborate on this item by providing additional information not in the ms, or briefly explain why the item is not applicable/relevant for your study

The app provided feedback on achievement of their goal and feedback on the overall nutrient reduction achieved through making swaps.

5-xii) Describe any co-interventions (incl. training/support)

Describe any co-interventions (incl. training/support): Clearly state any interventions that are provided in addition to the targeted eHealth intervention, as ehealth intervention may not be designed as stand-alone intervention. This includes training sessions and support [1]. It may be necessary to distinguish between the level of training required for the trial, and the level of training for a routine application outside of a RCT setting (discuss under item 21 – generalizability.

subitem not at all important

1 (selected)

2

3

4

5

essential

Clear selection

Does your paper address subitem 5-xii? *

Copy and paste relevant sections from the manuscript (include quotes in quotation marks "like this" to indicate direct quotes from your manuscript), or elaborate on this item by providing additional information not in the ms, or briefly explain why the item is not applicable/relevant for your study

none

Your answer must have a minimum of 25 characters.

6a) Completely defined pre-specified primary and secondary outcome measures, including how and when they were assessed

Does your paper address CONSORT subitem 6a? *

Copy and paste relevant sections from the manuscript (include quotes in quotation marks "like this" to indicate direct quotes from your manuscript), or elaborate on this item by providing additional information not in the ms, or briefly explain why the item is not applicable/relevant for your study

Outcome measures
Primary Outcomes
The primary objective of this study was to determine the feasibility of a larger study to evaluate the effectiveness of an app that offers healthier swaps while grocery shopping to improve the nutritional quality of food purchasing with respect to sugar, salt or saturated fat. The primary outcomes were pre-specified progression criteria as follows:
1) App use: at least 70% of participants in the active intervention groups use the app to obtain swaps on at least one occasion by the end of the second week after randomisation.
2) Follow up rate: at least 60% of participants in total (intervention and control) complete follow-up by scanning all their purchases for a minimum of 2 weeks over the entire follow up period (4 weeks).
Secondary outcomes
Feasibility outcomes included: 1) Recruitment rates: total recruited (including number signed up, eligible, consented, and randomised); 2) Period of time needed for recruitment of the final sample; 3) Outcome reporting: number of participants who fail to scan their purchases for a minimum of 2 weeks during follow up; number of participants who fail to complete the end-of-study questionnaires.
Process evaluation and qualitative outcome measures
A summary of app-related usage (within-app automatic recording) and acceptability measures collected through the end-of-study questionnaires at follow up: 1) Average number of shopping trips where the app was used to scan products in order to obtain a swap per week; 2) Number of occasions the app was used to scan products for a swap per trip; 3) Number of swaps made overall per week and per shopping trip; 4) Nutrient (SFA, sugar, and salt) reduction per swap made; 5) Use of specific functionality e.g. goal setting, feedback; 6) End-of-study questionnaires with free text to understand app usage and acceptability of the swaps; app functionality (e.g. if app scans the majority of products) and if this prompted other behaviours such as reading nutrition labels.

Exploratory effectiveness outcomes
Measures included changes in the nutrient content (SFA and sugar in g per 100 g) of household food purchases recorded in the app, between baseline and follow up in the intervention group compared to control.

6a-i) Online questionnaires: describe if they were validated for online use and apply CHERRIES items to describe how the questionnaires were designed/deployed

If outcomes were obtained through online questionnaires, describe if they were validated for online use and apply CHERRIES items to describe how the questionnaires were designed/deployed [9].

subitem not at all important

- 1 (selected)
- 2
- 3
- 4
- 5

essential

Clear selection

Does your paper address subitem 6a-i?

Copy and paste relevant sections from manuscript text

Your answer

6a-ii) Describe whether and how “use” (including intensity of use/dosage) was defined/measured/monitored

Describe whether and how “use” (including intensity of use/dosage) was defined/measured/monitored (logins, logfile analysis, etc.). Use/adoption metrics are important process outcomes that should be reported in any ehealth trial.

subitem not at all important

1 (selected)

2

3

4

5

essential

Clear selection

Does your paper address subitem 6a-ii?

Copy and paste relevant sections from manuscript text

Your answer

6a-iii) Describe whether, how, and when qualitative feedback from participants was obtained

Describe whether, how, and when qualitative feedback from participants was obtained (e.g., through emails, feedback forms, interviews, focus groups).

subitem not at all important

1 ○

2 ○

3 ○

4 ○

5 ◉

essential

Clear selection

Does your paper address subitem 6a-iii?

Copy and paste relevant sections from manuscript text

A summary of app-related usage (within-app automatic recording) and acceptability measures collected through the end-of-study questionnaires at follow up: 1) Average number of shopping trips where the app was used to scan products in order to obtain a swap per week; 2) Number of occasions the app was used to scan products for a swap per trip; 3) Number of swaps made overall per week and per shopping trip; 4) Nutrient (SFA, sugar, and salt) reduction per swap made; 5) Use of specific functionality e.g. goal setting, feedback; 6) End-of-study questionnaires with free text to understand app usage and acceptability of the swaps; app functionality (e.g. if app scans the majority of products) and if this prompted other behaviours such as reading nutrition labels.

6b) Any changes to trial outcomes after the trial commenced, with reasons

Does your paper address CONSORT subitem 6b? *

Copy and paste relevant sections from the manuscript (include quotes in quotation marks "like this" to indicate direct quotes from your manuscript), or elaborate on this item by providing additional information not in the ms, or briefly explain why the item is not applicable/relevant for your study

No changes to trial outcomes after the trial commenced

7a) How sample size was determined

NPT: When applicable, details of whether and how the clustering by care provides or centers was addressed

7a-i) Describe whether and how expected attrition was taken into account when calculating the sample size

Describe whether and how expected attrition was taken into account when calculating the sample size.

subitem not at all important

1 ○

2 ○

3 ○

4 ○

5 ◉

essential

Clear selection

Does your paper address subitem 7a-i?

Copy and paste relevant sections from manuscript title (include quotes in quotation marks "like this" to indicate direct quotes from your manuscript), or elaborate on this item by providing additional information not in the ms, or briefly explain why the item is not applicable/relevant for your study

This study was planned as a feasibility study. A sample size of 120 (n~30 per intervention group and n~10 per control group) was pre-specified as sufficient to enable testing of the trial methodology and outcome measures using parametric statistical models. The protocol was prospectively registered at ISRCTN13022312.

7b) When applicable, explanation of any interim analyses and stopping guidelines

Does your paper address CONSORT subitem 7b? *

Copy and paste relevant sections from the manuscript (include quotes in quotation marks "like this" to indicate direct quotes from your manuscript), or elaborate on this item by providing additional information not in the ms, or briefly explain why the item is not applicable/relevant for your study

No interim analyses and stopping guidelines were used

8a) Method used to generate the random allocation sequence

NPT: When applicable, how care providers were allocated to each trial group

Does your paper address CONSORT subitem 8a? *

Copy and paste relevant sections from the manuscript (include quotes in quotation marks "like this" to indicate direct quotes from your manuscript), or elaborate on this item by providing additional information not in the ms, or briefly explain why the item is not applicable/relevant for your study

After the 2-week run in period, participants were individually randomised to either an intervention arm or control following a 3:1 ratio (intervention: control) within each strata depending on their chosen nutrient of concern (sugar, salt or SFA). Participants who did not complete the baseline data collection were not randomised. Randomisation was stratified by the nutrient of concern using block randomisation with block sizes of 4 and 8.

8b) Type of randomisation; details of any restriction (such as blocking and block size)

Does your paper address CONSORT subitem 8b? *

Copy and paste relevant sections from the manuscript (include quotes in quotation marks "like this" to indicate direct quotes from your manuscript), or elaborate on this item by providing additional information not in the ms, or briefly explain why the item is not applicable/relevant for your study

After the 2-week run in period, participants were individually randomised to either an intervention arm or control following a 3:1 ratio (intervention: control) within each strata depending on their chosen nutrient of concern (sugar, salt or SFA). Participants who did not complete the baseline data collection were not randomised.

9) Mechanism used to implement the random allocation sequence (such as sequentially numbered containers), describing any steps taken to conceal the sequence until interventions were assigned

Does your paper address CONSORT subitem 9? *

Copy and paste relevant sections from the manuscript (include quotes in quotation marks "like this" to indicate direct quotes from your manuscript), or elaborate on this item by providing additional information not in the ms, or briefly explain why the item is not applicable/relevant for your study

Randomisation was performed by computer using a random allocation sequence (concealed to the investigators) and was stratified by the nutrient of concern using block randomisation with block sizes of 4 and 8.

10) Who generated the random allocation sequence, who enrolled participants, and who assigned participants to interventions

Does your paper address CONSORT subitem 10? *

Copy and paste relevant sections from the manuscript (include quotes in quotation marks "like this" to indicate direct quotes from your manuscript), or elaborate on this item by providing additional information not in the ms, or briefly explain why the item is not applicable/relevant for your study

It was not possible to blind the participants to the intervention due to the nature of the intervention, but randomisation was performed remotely via computer-generated randomisation and the researchers were not aware of the treatment group until after randomisation was complete.

11a) If done, who was blinded after assignment to interventions (for example, participants, care providers, those assessing outcomes) and how

NPT: Whether or not administering co-interventions were blinded to group assignment

11a-i) Specify who was blinded, and who wasn't

Specify who was blinded, and who wasn't. Usually, in web-based trials it is not possible to blind the participants [1, 3] (this should be clearly acknowledged), but it may be possible to blind outcome assessors, those doing data analysis or those administering co-interventions (if any).

subitem not at all important

- 1 (selected)
- 2
- 3
- 4
- 5

essential

Clear selection

Does your paper address subitem 11a-i? *

Copy and paste relevant sections from the manuscript (include quotes in quotation marks "like this" to indicate direct quotes from your manuscript), or elaborate on this item by providing additional information not in the ms, or briefly explain why the item is not applicable/relevant for your study

It was not possible to blind the participants to the intervention due to the nature of the intervention, but randomisation was performed remotely via computer-generated randomisation and the researchers were not aware of the treatment group until after randomisation was complete.

11a-ii) Discuss e.g., whether participants knew which intervention was the “intervention of interest” and which one was the “comparator”

Informed consent procedures (4a-ii) can create biases and certain expectations - discuss e.g., whether participants knew which intervention was the “intervention of interest” and which one was the “comparator”.

subitem not at all important

- 1 (selected)
- 2
- 3
- 4
- 5

essential

Clear selection

Does your paper address subitem 11a-ii?

Copy and paste relevant sections from the manuscript (include quotes in quotation marks "like this" to indicate direct quotes from your manuscript), or elaborate on this item by providing additional information not in the ms, or briefly explain why the item is not applicable/relevant for your study

Participants were not aware of the intervention of interest

11b) If relevant, description of the similarity of interventions

(this item is usually not relevant for ehealth trials as it refers to similarity of a placebo or sham intervention to a active medication/intervention)

Does your paper address CONSORT subitem 11b? *

Copy and paste relevant sections from the manuscript (include quotes in quotation marks "like this" to indicate direct quotes from your manuscript), or elaborate on this item by providing additional information not in the ms, or briefly explain why the item is not applicable/relevant for your study

The interventions were not similar

12a) Statistical methods used to compare groups for primary and secondary outcomes

NPT: When applicable, details of whether and how the clustering by care providers or centers was addressed

Does your paper address CONSORT subitem 12a? *

Copy and paste relevant sections from the manuscript (include quotes in quotation marks "like this" to indicate direct quotes from your manuscript), or elaborate on this item by providing additional information not in the ms, or briefly explain why the item is not applicable/relevant for your study

Baseline characteristics overall and by trial arm were summarised as means and standard deviations for continuous variables and counts and percentages for categorical variables. Baseline characteristics were coded as: age (years); gender (men, women, other/not specified); ethnic group (white, Black, Asian, Mixed, Other); education (no formal qualifications, secondary education, higher education); income (>£15,000, £15,000-£24,999, £25,000-£39,999, £40,000-£75,000, >£75,000); household size (1, 2-4, 5+); grocery shopping habits (e.g. spending >£25 on groceries >once a week, once a week, once a fortnight, once a month, <once a month); and relevant self-reported health history (e.g. concerns related to weight, high blood pressure, diabetes, heart disease).
Descriptive statistics were used to present the primary and secondary outcomes using all data available, regardless of whether participants completed the trial or withdrew.
For the exploratory effectiveness measures we used data from products with available information on weight or volume as well as the nutrient content (g per 100g) to estimate changes in SFA and sugar for food purchases recorded in the app. Excluded items in this analysis included those categorised as fresh fruits, vegetables or with no/minimal nutrient content (e.g. sugar free gum) as well as products that were manually entered in the app and/or those missing nutrient/volume information. We used linear regression models to investigate changes in the nutrient content of food purchases between baseline and follow up in the intervention vs control group. Tests for the linear regression assumptions were conducted and met. The main models included adjustment for baseline values of the dependent variable. Potential confounding due to imbalance in baseline characteristics was investigated and the variables were adjusted in sensitivity analyses. StataSE 16 was used for these analyses. A P<0.05 was set to denote statistical significance.
A descriptive analysis of the qualitative outcome measures was conducted using the Braun and Clarke's method for thematic analysis in NVivo 1 software (27). Each response was line-by-line coded and codes were inductively constructed based on the study aim. We then organised codes into sub-themes using the One Sheet of Paper technique and produced top-level themes (28).

12a-i) Imputation techniques to deal with attrition / missing values

Imputation techniques to deal with attrition / missing values: Not all participants will use the intervention/comparator as intended and attrition is typically high in ehealth trials. Specify how participants who did not use the application or dropped out from the trial were treated in the statistical analysis (a complete case analysis is strongly discouraged, and simple imputation techniques such as LOCF may also be problematic [4]).

subitem not at all important

1 ○

2 ○

3 ◉

4 ○

5 ○

essential

Clear selection

Does your paper address subitem 12a-i? *

Copy and paste relevant sections from the manuscript (include quotes in quotation marks "like this" to indicate direct quotes from your manuscript), or elaborate on this item by providing additional information not in the ms, or briefly explain why the item is not applicable/relevant for your study

Descriptive statistics were used to present the primary and secondary outcomes using all data available, regardless of whether participants completed the trial or withdrew.

12b) Methods for additional analyses, such as subgroup analyses and adjusted analyses

Does your paper address CONSORT subitem 12b? *

Copy and paste relevant sections from the manuscript (include quotes in quotation marks "like this" to indicate direct quotes from your manuscript), or elaborate on this item by providing additional information not in the ms, or briefly explain why the item is not applicable/relevant for your study

Potential confounding due to imbalance in baseline characteristics was investigated and the variables were adjusted in sensitivity analyses.

X26) REB/IRB Approval and Ethical Considerations [recommended as subheading under "Methods"] (not a CONSORT item)

X26-i) Comment on ethics committee approval

subitem not at all important

1 ○

2 ○

3 ○

4 ○

5 ◉

essential

Clear selection

Does your paper address subitem X26-i?

Copy and paste relevant sections from the manuscript (include quotes in quotation marks "like this" to indicate direct quotes from your manuscript), or elaborate on this item by providing additional information not in the ms, or briefly explain why the item is not applicable/relevant for your study

This study was reviewed and approved by the University of Oxford Medical Sciences Interdivisional Research Ethics Committee (Ref R67216/RE001).

x26-ii) Outline informed consent procedures

Outline informed consent procedures e.g., if consent was obtained offline or online (how? Checkbox, etc.?), and what information was provided (see 4a-ii). See [6] for some items to be included in informed consent documents.

subitem not at all important

1 (selected)

2

3

4

5

essential

Clear selection

Does your paper address subitem X26-ii?

Copy and paste relevant sections from the manuscript (include quotes in quotation marks "like this" to indicate direct quotes from your manuscript), or elaborate on this item by providing additional information not in the ms, or briefly explain why the item is not applicable/relevant for your study

After giving written informed consent to participate in the study and completing screening and baseline assessment, participants entered a 2-week run-in period where they used a basic version of the SwapSHOP app (no swap/behavioural functionality shown) to record their grocery shopping.

X26-iii) Safety and security procedures

Safety and security procedures, incl. privacy considerations, and any steps taken to reduce the likelihood or detection of harm (e.g., education and training, availability of a hotline)

subitem not at all important

1 (selected)

2

3

4

5

essential

Clear selection

Does your paper address subitem X26-iii?

Copy and paste relevant sections from the manuscript (include quotes in quotation marks "like this" to indicate direct quotes from your manuscript), or elaborate on this item by providing additional information not in the ms, or briefly explain why the item is not applicable/relevant for your study

Your answer

RESULTS

13a) For each group, the numbers of participants who were randomly assigned, received intended treatment, and were analysed for the primary outcome

NPT: The number of care providers or centers performing the intervention in each group and the number of patients treated by each care provider in each center

Does your paper address CONSORT subitem 13a? *

Copy and paste relevant sections from the manuscript (include quotes in quotation marks "like this" to indicate direct quotes from your manuscript), or elaborate on this item by providing additional information not in the ms, or briefly explain why the item is not applicable/relevant for your study

Study participants were recruited between April and October 2021. Of the 289 interested participants, 190 were eligible, provided consent and entered the 2-week run-in period, of which 112 (39%) successfully completed the task and were randomised (Figure 1). Fifty-one were randomised to the SFA group (n=38 to intervention; n=13 to control), 55 participants were randomised to the sugar group (n=40 to intervention; n=15 to control); and six to the salt group (n=5 to intervention; n=1 to control). Of all randomised participants, n=100 (89%) completed the study and were analysed as follows: n=49 (96%) in the SFA group; n=51 (93%) in the sugar group. In the SFA group n=2 and in the sugar group n=4 were withdrawn from the study and excluded from analysis because their data indicated fraudulent activity (e.g. fake phone numbers and implausible shopping patterns). Data from the six participants that were randomised to the salt group were not analysed as this group did not reach the target sample by the end of the recruitment period.

13b) For each group, losses and exclusions after randomisation, together with reasons

Does your paper address CONSORT subitem 13b? (NOTE: Preferably, this is shown in a CONSORT flow diagram) *

Copy and paste relevant sections from the manuscript (include quotes in quotation marks "like this" to indicate direct quotes from your manuscript), or elaborate on this item by providing additional information not in the ms, or briefly explain why the item is not applicable/relevant for your study

Study participants were recruited between April and October 2021. Of the 289 interested participants, 190 were eligible, provided consent and entered the 2-week run-in period, of which 112 (39%) successfully completed the task and were randomised (Figure 1). Fifty-one were randomised to the SFA group (n=38 to intervention; n=13 to control), 55 participants were randomised to the sugar group (n=40 to intervention; n=15 to control); and six to the salt group (n=5 to intervention; n=1 to control). Of all randomised participants, n=100 (89%) completed the study and were analysed as follows: n=49 (96%) in the SFA group; n=51 (93%) in the sugar group. In the SFA group n=2 and in the sugar group n=4 were withdrawn from the study and excluded from analysis because their data indicated fraudulent activity (e.g. fake phone numbers and implausible shopping patterns). Data from the six participants that were randomised to the salt group were not analysed as this group did not reach the target sample by the end of the recruitment period.

13b-i) Attrition diagram

Strongly recommended: An attrition diagram (e.g., proportion of participants still logging in or using the intervention/comparator in each group plotted over time, similar to a survival curve) or other figures or tables demonstrating usage/dose/engagement.

subitem not at all important

- 1 ○
- 2 ○
- 3 ○
- 4 ○
- 5 ◉

essential

Clear selection

Does your paper address subitem 13b-i?

Copy and paste relevant sections from the manuscript or cite the figure number if applicable (include quotes in quotation marks "like this" to indicate direct quotes from your manuscript), or elaborate on this item by providing additional information not in the ms, or briefly explain why the item is not applicable/relevant for your study

Figure 1 shows the attrition diagram

14a) Dates defining the periods of recruitment and follow-up

Does your paper address CONSORT subitem 14a? *

Copy and paste relevant sections from the manuscript (include quotes in quotation marks "like this" to indicate direct quotes from your manuscript), or elaborate on this item by providing additional information not in the ms, or briefly explain why the item is not applicable/relevant for your study

Study participants were recruited between April and October 2021.

14a-i) Indicate if critical “secular events” fell into the study period

Indicate if critical “secular events” fell into the study period, e.g., significant changes in Internet resources available or “changes in computer hardware or Internet delivery resources”

subitem not at all important

- 1 (selected)
- 2
- 3
- 4
- 5

essential

Clear selection

Does your paper address subitem 14a-i?

Copy and paste relevant sections from the manuscript (include quotes in quotation marks "like this" to indicate direct quotes from your manuscript), or elaborate on this item by providing additional information not in the ms, or briefly explain why the item is not applicable/relevant for your study

Your answer

14b) Why the trial ended or was stopped (early)

Does your paper address CONSORT subitem 14b? *

Copy and paste relevant sections from the manuscript (include quotes in quotation marks "like this" to indicate direct quotes from your manuscript), or elaborate on this item by providing additional information not in the ms, or briefly explain why the item is not applicable/relevant for your study

This trial was not stopped earlier

15) A table showing baseline demographic and clinical characteristics for each group

NPT: When applicable, a description of care providers (case volume, qualification, expertise, etc.) and centers (volume) in each group

Does your paper address CONSORT subitem 15? *

Copy and paste relevant sections from the manuscript (include quotes in quotation marks "like this" to indicate direct quotes from your manuscript), or elaborate on this item by providing additional information not in the ms, or briefly explain why the item is not applicable/relevant for your study

table 1 shows the baseline demographic characteristics

15-i) Report demographics associated with digital divide issues

In ehealth trials it is particularly important to report demographics associated with digital divide issues, such as age, education, gender, social-economic status, computer/Internet/ehealth literacy of the participants, if known.

subitem not at all important

1 (selected)

2

3

4

5

essential

Clear selection

Does your paper address subitem 15-i? *

Copy and paste relevant sections from the manuscript (include quotes in quotation marks "like this" to indicate direct quotes from your manuscript), or elaborate on this item by providing additional information not in the ms, or briefly explain why the item is not applicable/relevant for your study

table 1 shows the baseline demographic characteristics

16) For each group, number of participants (denominator) included in each analysis and whether the analysis was by original assigned groups

16-i) Report multiple “denominators” and provide definitions

Report multiple “denominators” and provide definitions: Report N’s (and effect sizes) “across a range of study participation [and use] thresholds” [1], e.g., N exposed, N consented, N used more than x times, N used more than y weeks, N participants “used” the intervention/comparator at specific pre-defined time points of interest (in absolute and relative numbers per group). Always clearly define “use” of the intervention.

subitem not at all important

1 ○

2 ○

3 ○

4 ○

5 ◉

essential

Clear selection

Does your paper address subitem 16-i? *

Copy and paste relevant sections from the manuscript (include quotes in quotation marks "like this" to indicate direct quotes from your manuscript), or elaborate on this item by providing additional information not in the ms, or briefly explain why the item is not applicable/relevant for your study

The number of participants recruited for the salt group did not reach the target sample size, despite additional recruitment attempts, suggesting a full-scale trial for salt reduction would not be feasible. Data from participants in this group was not analysed further.
For participants randomised to the SFA and sugar, the two progression criteria were met above the set thresholds (Table 2).

16-ii) Primary analysis should be intent-to-treat

Primary analysis should be intent-to-treat, secondary analyses could include comparing only “users”, with the appropriate caveats that this is no longer a randomized sample (see 18-i).

subitem not at all important

1 ○

2 ○

3 ○

4 ○

5 ◉

essential

Clear selection

Does your paper address subitem 16-ii?

Copy and paste relevant sections from the manuscript (include quotes in quotation marks "like this" to indicate direct quotes from your manuscript), or elaborate on this item by providing additional information not in the ms, or briefly explain why the item is not applicable/relevant for your study

Overall, most intervention participants (81% in the SFA group and 87% in the sugar group) used the app to obtain swaps on at least 1 occasion by the end of the 2nd week after randomisation. In addition, 89% of intervention participants and 96% of control participants completed follow-up by scanning all purchases for a minimum of 2 weeks over the entire follow up period.

17a) For each primary and secondary outcome, results for each group, and the estimated effect size and its precision (such as 95% confidence interval)

Does your paper address CONSORT subitem 17a? *

Copy and paste relevant sections from the manuscript (include quotes in quotation marks "like this" to indicate direct quotes from your manuscript), or elaborate on this item by providing additional information not in the ms, or briefly explain why the item is not applicable/relevant for your study

Not applicable as this is a feasibility study

17a-i) Presentation of process outcomes such as metrics of use and intensity of use

In addition to primary/secondary (clinical) outcomes, the presentation of process outcomes such as metrics of use and intensity of use (dose, exposure) and their operational definitions is critical. This does not only refer to metrics of attrition (13-b) (often a binary variable), but also to more continuous exposure metrics such as “average session length”. These must be accompanied by a technical description how a metric like a “session” is defined (e.g., timeout after idle time) [1] (report under item 6a).

subitem not at all important

- 1 ○
- 2 ○
- 3 ○
- 4 ○
- 5 ◉

essential

Clear selection

Does your paper address subitem 17a-i?

Copy and paste relevant sections from the manuscript (include quotes in quotation marks "like this" to indicate direct quotes from your manuscript), or elaborate on this item by providing additional information not in the ms, or briefly explain why the item is not applicable/relevant for your study

Participants randomised to the SwapSHOP intervention used the app regularly (Table 3). The average number of shopping trips where the app was used to obtain a swap was 5 in the SFA group (92% of shopping trips) and 5.4 in the sugar group (83% of shopping trips). The average number of occasions where the app was used to scan products for a swap in each shopping trip was 2.5 times in the SFA group and 3.3 in the sugar group. Overall, participants set goals averaging around 2 swaps per shopping trip. Results showed that individual product swaps were associated with an average reduction in total sugars (-12.5 g of sugar per 100g SD 6.97) or in SFA (-4 g of SFA per 100g SD 2.16) in the sugar and SFA groups respectively.

17b) For binary outcomes, presentation of both absolute and relative effect sizes is recommended

Does your paper address CONSORT subitem 17b? *

Copy and paste relevant sections from the manuscript (include quotes in quotation marks "like this" to indicate direct quotes from your manuscript), or elaborate on this item by providing additional information not in the ms, or briefly explain why the item is not applicable/relevant for your study

No binary outcomes are presented

18) Results of any other analyses performed, including subgroup analyses and adjusted analyses, distinguishing pre-specified from exploratory

Does your paper address CONSORT subitem 18? *

Copy and paste relevant sections from the manuscript (include quotes in quotation marks "like this" to indicate direct quotes from your manuscript), or elaborate on this item by providing additional information not in the ms, or briefly explain why the item is not applicable/relevant for your study

Exploratory effectiveness outcomes
Baseline and follow up data on food purchases with available information on volume and nutrient content were available for 86% of participants in the sugar group and 82% of participants in the SFA group. This analysis included all food purchases recorded in the app but excluded purchases that were entered manually (22%) or those with no/minimal/missing nutritional information (16%). The degree of missingness in the food purchasing data was comparable between groups (manually entered products: 23% intervention vs 19% control group; missing nutrient information: 15% intervention vs 16% control group).
There was evidence of changes in the intended direction in both intervention groups (Table 4). The sugar group reduced total sugars in their grocery purchases by -1 (95%CI -1.97, -0.03) g/100g, while the control group was 0.32 (-1.47, 2.11) g/100g, though the differences between groups were not significant (-0.68 (95%CI -1.94, 0.58), g/100g, p=0.282 adjusted for baseline values). The SFA group reduced total SFA in food purchases by -0.56 (95%CI -1.02, -0.19) g/100g, and the control group increased by 0.52 (95%CI -0.19, 1.22) g/100g, with a significant between group difference of -1.05 (96%CI -1.83, -0.27), g/100g, p=0.009 adjusted for baseline values. These results were robust in sensitivity analyses adjusted for age, sex, ethnicity, and income (Multimedia Appendix 1 - Table 2).

18-i) Subgroup analysis of comparing only users

A subgroup analysis of comparing only users is not uncommon in ehealth trials, but if done, it must be stressed that this is a self-selected sample and no longer an unbiased sample from a randomized trial (see 16-iii).

subitem not at all important

1 (selected)

2

3

4

5

essential

Clear selection

Does your paper address subitem 18-i?

Copy and paste relevant sections from the manuscript (include quotes in quotation marks "like this" to indicate direct quotes from your manuscript), or elaborate on this item by providing additional information not in the ms, or briefly explain why the item is not applicable/relevant for your study

No subgroups analyses were performed

19) All important harms or unintended effects in each group

(for specific guidance see CONSORT for harms)

Does your paper address CONSORT subitem 19? *

Copy and paste relevant sections from the manuscript (include quotes in quotation marks "like this" to indicate direct quotes from your manuscript), or elaborate on this item by providing additional information not in the ms, or briefly explain why the item is not applicable/relevant for your study

no harms were expected or recorded

19-i) Include privacy breaches, technical problems

Include privacy breaches, technical problems. This does not only include physical “harm” to participants, but also incidents such as perceived or real privacy breaches [1], technical problems, and other unexpected/unintended incidents. “Unintended effects” also includes unintended positive effects [2].

subitem not at all important

1 (selected)

2

3

4

5

essential

Clear selection

Does your paper address subitem 19-i?

Copy and paste relevant sections from the manuscript (include quotes in quotation marks "like this" to indicate direct quotes from your manuscript), or elaborate on this item by providing additional information not in the ms, or briefly explain why the item is not applicable/relevant for your study

Your answer

19-ii) Include qualitative feedback from participants or observations from staff/researchers

Include qualitative feedback from participants or observations from staff/researchers, if available, on strengths and shortcomings of the application, especially if they point to unintended/unexpected effects or uses. This includes (if available) reasons for why people did or did not use the application as intended by the developers.

subitem not at all important

1 ○

2 ○

3 ○

4 ○

5 ◉

essential

Clear selection

Does your paper address subitem 19-ii?

Copy and paste relevant sections from the manuscript (include quotes in quotation marks "like this" to indicate direct quotes from your manuscript), or elaborate on this item by providing additional information not in the ms, or briefly explain why the item is not applicable/relevant for your study

The results of the qualitative research relating to the acceptability of the app, the feedback and swaps provided through the app as well as the usefulness and comprehensiveness of the app were summarised into four themes (Multimedia Appendix 1 - Table 1). Overall, most participants shared positive experiences of the intervention, noting that the app was helpful and the swap suggestions were acceptable. They valued the novelty of the app and the traffic light food labelling system, which encouraged them to read food labels. The key barriers to usability were that some grocery stores were not supported by the app and poor product coverage within the app database. Though participants could manually enter their food purchases to find alternatives, they reported this was time-consuming and a barrier to engagement. Some participants also found it hard to locate the suggested swaps in the store, or were frustrated that the suggested swaps were not available in the store or that the product database in the app did not include own-label products in some stores. A key barrier to the acceptability of the intervention was the specificity of some swap suggestions, which were neither like-for-like, nor considered personal/household dietary preferences and meant that shoppers could not act on the prompt. In other instances, some participants found it challenging to reduce one nutritional component if it involved an increase in another and they expressed a desire for a swap suggestion that recognised the overall healthfulness of the product. Participants suggested that more information on the fibre composition of swaps, recommended portion sizes and price comparison would inform their purchasing behaviour. Most participants noted that other visual self-monitoring techniques would improve engagement with the intervention.

DISCUSSION

22) Interpretation consistent with results, balancing benefits and harms, and considering other relevant evidence

NPT: In addition, take into account the choice of the comparator, lack of or partial blinding, and unequal expertise of care providers or centers in each group

22-i) Restate study questions and summarize the answers suggested by the data, starting with primary outcomes and process outcomes (use)

Restate study questions and summarize the answers suggested by the data, starting with primary outcomes and process outcomes (use).

subitem not at all important

1 ○

2 ○

3 ○

4 ○

5 ◉

essential

Clear selection

Does your paper address subitem 22-i? *

Copy and paste relevant sections from the manuscript (include quotes in quotation marks "like this" to indicate direct quotes from your manuscript), or elaborate on this item by providing additional information not in the ms, or briefly explain why the item is not applicable/relevant for your study

Participants in this study were willing and able to use the SwapSHOP app as intended, and the research methods ran as planned with high levels of adherence and follow up recorded. It provided preliminary evidence of effectiveness to support dietary change to lower intake of sugars and SFAs. There was little evidence that this general population sample were motivated to reduce salt intake using the SwapSHOP app. Further improvements to the app, especially enhancing the coverage of product data and the specificity of the swap algorithm are needed to provide a higher quality user experience.

22-ii) Highlight unanswered new questions, suggest future research

Highlight unanswered new questions, suggest future research.

subitem not at all important

1 ○

2 ○

3 ○

4 ○

5 ◉

essential

Clear selection

Does your paper address subitem 22-ii?

Copy and paste relevant sections from the manuscript (include quotes in quotation marks "like this" to indicate direct quotes from your manuscript), or elaborate on this item by providing additional information not in the ms, or briefly explain why the item is not applicable/relevant for your study

Although the SwapSHOP app included the major UK grocery retailers, participants particularly reported issues related to the product database; the limited range of UK grocery stores, and the improvements needed in the swapping function to create a list of healthier options that are similar to the original product and to accommodate dietary preferences. These aspects need to be addressed in any future version of the app to maximise usability and acceptability.

20) Trial limitations, addressing sources of potential bias, imprecision, and, if relevant, multiplicity of analyses

20-i) Typical limitations in ehealth trials

Typical limitations in ehealth trials: Participants in ehealth trials are rarely blinded. Ehealth trials often look at a multiplicity of outcomes, increasing risk for a Type I error. Discuss biases due to non-use of the intervention/usability issues, biases through informed consent procedures, unexpected events.

subitem not at all important

1 ○

2 ○

3 ○

4 ○

5 ◉

essential

Clear selection

Does your paper address subitem 20-i? *

Copy and paste relevant sections from the manuscript (include quotes in quotation marks "like this" to indicate direct quotes from your manuscript), or elaborate on this item by providing additional information not in the ms, or briefly explain why the item is not applicable/relevant for your study

Although the SwapSHOP app included the major UK grocery retailers, participants particularly reported issues related to the product database; the limited range of UK grocery stores, and the improvements needed in the swapping function to create a list of healthier options that are similar to the original product and to accommodate dietary preferences. These aspects need to be addressed in any future version of the app to maximise usability and acceptability. Purchased products that were recorded manually by the participants, or were missing nutrient information were excluded from the analysis of changes in total purchases, limiting the robustness of the exploratory effectiveness outcome results, though this should not differ by trial arm.

21) Generalisability (external validity, applicability) of the trial findings

NPT: External validity of the trial findings according to the intervention, comparators, patients, and care providers or centers involved in the trial

21-i) Generalizability to other populations

Generalizability to other populations: In particular, discuss generalizability to a general Internet population, outside of a RCT setting, and general patient population, including applicability of the study results for other organizations

subitem not at all important

1 ○

2 ○

3 ●

4 ○

5 ○

essential

Clear selection

Does your paper address subitem 21-i?

Copy and paste relevant sections from the manuscript (include quotes in quotation marks "like this" to indicate direct quotes from your manuscript), or elaborate on this item by providing additional information not in the ms, or briefly explain why the item is not applicable/relevant for your study

Another limitation is that the study recruited a small, self-selected sample of people, motivated to take action to improve their diet quality. A large proportion of participants reported receiving higher education and living in less deprived geographical areas than the national average (40). A lower socioeconomic status is related to poorer dietary quality and health outcomes, hence the observed results may not represent the wider population with lower adherence to dietary recommendations (41). Ethnicity is also related to food choice (42) and although this study mostly included people of white backgrounds, the app database included a wide range of products available in the UK market, therefore covering different dietary preferences that apply to other ethnic groups.

21-ii) Discuss if there were elements in the RCT that would be different in a routine application setting

Discuss if there were elements in the RCT that would be different in a routine application setting (e.g., prompts/reminders, more human involvement, training sessions or other co-interventions) and what impact the omission of these elements could have on use, adoption, or outcomes if the intervention is applied outside of a RCT setting.

subitem not at all important

- 1 (selected)
- 2
- 3
- 4
- 5

essential

Clear selection

Does your paper address subitem 21-ii?

Copy and paste relevant sections from the manuscript (include quotes in quotation marks "like this" to indicate direct quotes from your manuscript), or elaborate on this item by providing additional information not in the ms, or briefly explain why the item is not applicable/relevant for your study

Your answer

OTHER INFORMATION

23) Registration number and name of trial registry

Does your paper address CONSORT subitem 23? *

Copy and paste relevant sections from the manuscript (include quotes in quotation marks "like this" to indicate direct quotes from your manuscript), or elaborate on this item by providing additional information not in the ms, or briefly explain why the item is not applicable/relevant for your study

This was a prospectively registered (ISRCTN13022312)

24) Where the full trial protocol can be accessed, if available

Does your paper address CONSORT subitem 24? *

Cite a Multimedia Appendix, other reference, or copy and paste relevant sections from the manuscript (include quotes in quotation marks "like this" to indicate direct quotes from your manuscript), or elaborate on this item by providing additional information not in the ms, or briefly explain why the item is not applicable/relevant for your study

The full protocol can be accessed upon request

25) Sources of funding and other support (such as supply of drugs), role of funders

Does your paper address CONSORT subitem 25? *

Copy and paste relevant sections from the manuscript (include quotes in quotation marks "like this" to indicate direct quotes from your manuscript), or elaborate on this item by providing additional information not in the ms, or briefly explain why the item is not applicable/relevant for your study

This study was funded by the NIHR Applied Research Collaborations Oxford. SJ, MN, AH were funded by the NIHR Oxford Biomedical Research Centre (BRC) Obesity, Diet and Lifestyle Theme. CP, GH and SJ were funded by the NIHR Applied Research Collaborations Oxford. CL is funded by the Engineering and Physical Sciences Research Council (EPSRC) and the NIHR Oxford BRC. SPR was supported by a British Heart Foundation Clinical Research Training Fellowship (FS/16/34/32211). The funders had no role in designing the study, data collection, analysis, interpretation of data, writing the report, or the decision to submit the report for publication.

X27) Conflicts of Interest (not a CONSORT item)

X27-i) State the relation of the study team towards the system being evaluated

In addition to the usual declaration of interests (financial or otherwise), also state the relation of the study team towards the system being evaluated, i.e., state if the authors/evaluators are distinct from or identical with the developers/sponsors of the intervention.

subitem not at all important

1 (selected)

2

3

4

5

essential

Clear selection

Does your paper address subitem X27-i?

Copy and paste relevant sections from the manuscript (include quotes in quotation marks "like this" to indicate direct quotes from your manuscript), or elaborate on this item by providing additional information not in the ms, or briefly explain why the item is not applicable/relevant for your study

Your answer

About the CONSORT EHEALTH checklist

As a result of using this checklist, did you make changes in your manuscript? *

- ( ) yes, major changes
- ( ) yes, minor changes
- (•) no

What were the most important changes you made as a result of using this checklist?

No major changes were done

How much time did you spend on going through the checklist INCLUDING making changes in your manuscript *

20 minutes going through the checklist

As a result of using this checklist, do you think your manuscript has improved? *

- (•) yes
- ( ) no
- ( ) Other:

Would you like to become involved in the CONSORT EHEALTH group?

This would involve for example becoming involved in participating in a workshop and writing an "Explanation and Elaboration" document

- ○ yes
- ◉ no
- ○ Other:

Clear selection

Any other comments or questions on CONSORT EHEALTH

Your answer

STOP - Save this form as PDF before you click submit

To generate a record that you filled in this form, we recommend to generate a PDF of this page (on a Mac, simply select "print" and then select "print as PDF") before you submit it.

When you submit your (revised) paper to JMIR, please upload the PDF as supplementary file.

Don't worry if some text in the textboxes is cut off, as we still have the complete information in our database. Thank you!

Final step: Click submit !

Click submit so we have your answers in our database!

Submit Clear form

Never submit passwords through Google Forms.

This content is neither created nor endorsed by Google. Report Abuse - Terms of Service - Privacy Policy

Google Forms
